# Supplementary material for: Spatially resolved phosphoproteomics reveals fibroblast growth factor receptor recycling-driven regulation of autophagy and survival
Source: Nat Commun. 2022 Nov 3;13:6589. doi: 10.1038/s41467-022-34298-2 (PMC9633600; doi:10.1038/s41467-022-34298-2)
Supplement: Supplementary file 1 — Supplementary information [file 41467_2022_34298_MOESM1_ESM.pdf]

Spatially Resolved Phosphoproteomics Reveals Fibroblast Growth Factor Receptor Recycling-driven Regulation of Autophagy and Survival

Supplementary Information

Figures

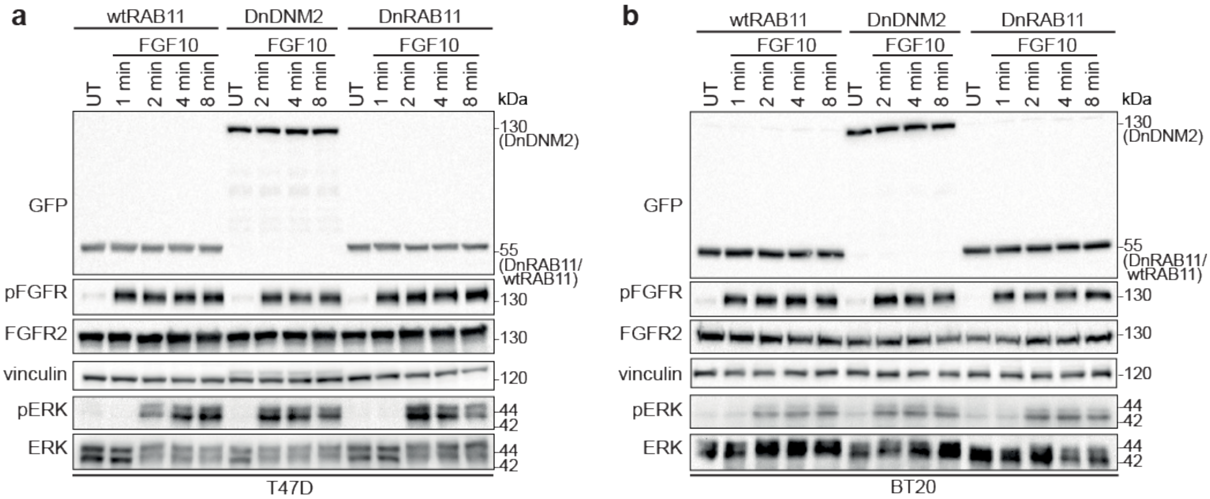

Supplementary Fig. 1. FGFR2b signalling in epithelial cells. Immunoblot analysis (N>=3 independent biological replicates) with the indicated antibodies of T47D (**a**) or BT20 (**b**) breast cancer epithelial cells expressing endogenous FGFR2b and transfected with wtRAB11, DnRAB11 or DnDNM2, and treated with vehicle (UT) or with FGF10 for 1, 2, 4 and 8 min. These time points represent early signalling activation. No significant changes in phosphorylation of FGFR and ERK were detected, consistent with the results observed in HeLa_FGFR2bST. Source data are provided as a Source Data file.

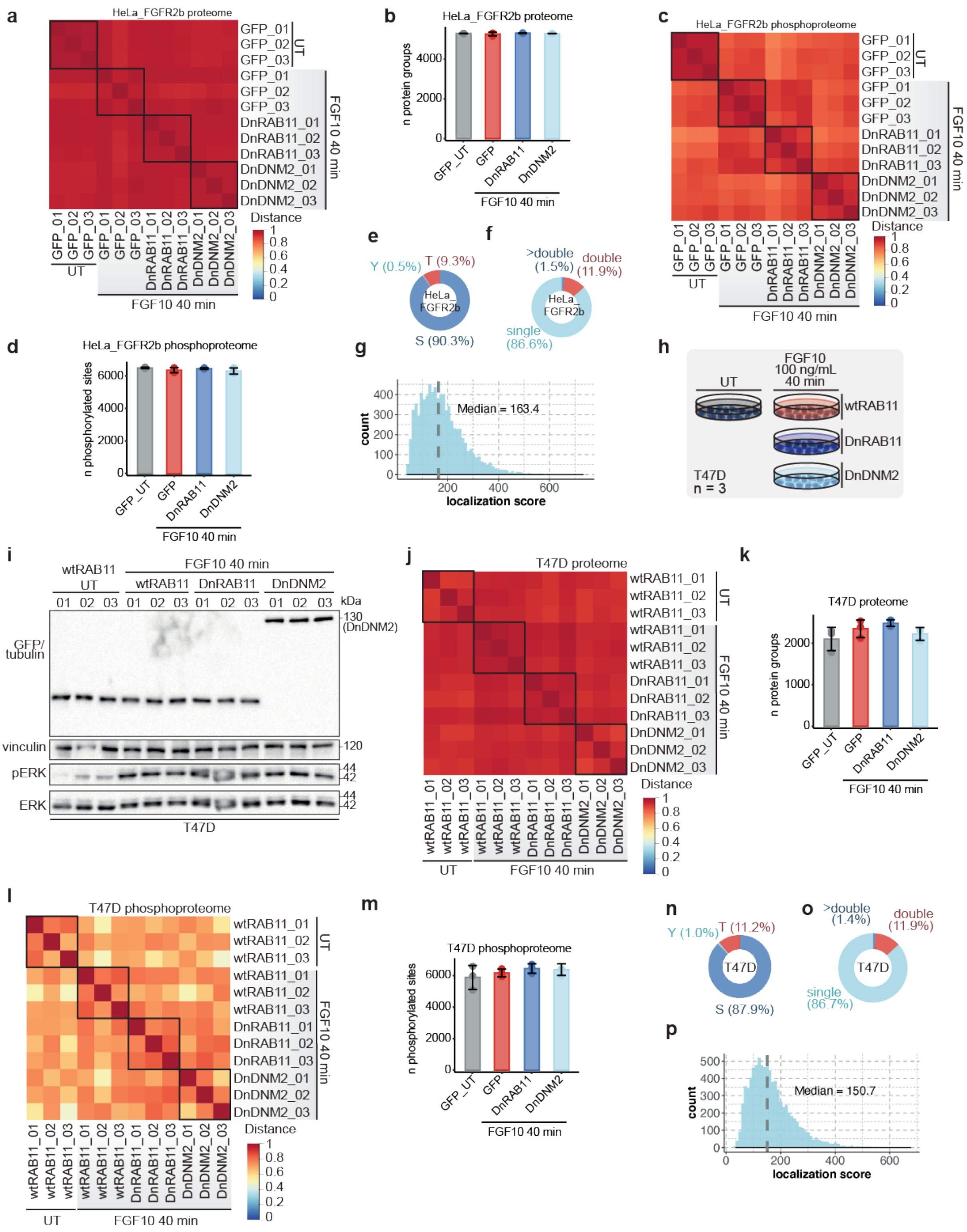

Supplementary Fig. 2. Quality assessment of the HeLa and T47D phosphoproteome and proteome. a Heat map of Pearson correlation of the HeLa proteome shows that transient expression (< 72h) of dominant negative constructs did not noticeably change the background proteome of HeLa cells. **b** Number (n) of protein groups quantified in each condition (Supplementary Data 1). Values represent mean ± SD of N = 3 independent biological

replicates. **c** Heat map of the Pearson correlation of the HeLa phosphoproteome shows good reproducibility and variation within experimental conditions. **d** Number (n) of phosphorylated peptides in each condition (Supplementary Data 2). Values represent mean ± SD of n = 3 independent biological replicates. **e** Distribution of peptides containing phosphorylated Serine (S), Threonine (T) and Tyrosine (Y) residues (%). **f** Distribution of peptides containing a single, double of more than double modification (%). **g** The distribution of phosphorylated peptides score showed that most of the peptides were identified with high Andromeda score (median: 163), consistent with[22]. **h** Workflow of the phosphoproteomics experiment in T47D cells transiently expressing either wtRAB11, DnRAB11 or DnDNM2 and treated with vehicle (UT) or with FGF10 for 40 min. **i** Immunoblot analysis (N>=3 independent biological replicates) with the indicated antibodies of T47D expressing either GFP, DnRAB11 or DnDNM2 and treated with vehicle (UT) or with FGF10 for 40 min. **j** Heat map of the Pearson correlation of the T47D proteome shows that transient expression (< 72h) of dominant negative constructs did not noticeably change the background proteome of T47D cells. **k** Number (n) of protein groups quantified in each condition (Supplementary Data 3). Values represent mean ± SD of N = 3 independent biological replicates. **l** Heat map of the Pearson correlation of the T47D phosphoproteome shows good reproducibility and variation within experimental conditions. **m** Number (n) of phosphorylated sites in each condition (Supplementary Data 4). Values represent mean ± SD of N= 3 independent biological replicates. **n** Distribution of peptides containing phosphorylated Serine (S), Threonine (T) and Tyrosine (Y) residues (%). **o** Distribution of peptides containing a single, double of more than double modification (%). **p** The distribution of phosphorylated peptides score showed that most of the peptides were identified with high Andromeda score (median: 150.7), consistent with previous[22]. Source data are provided as a Source Data file.

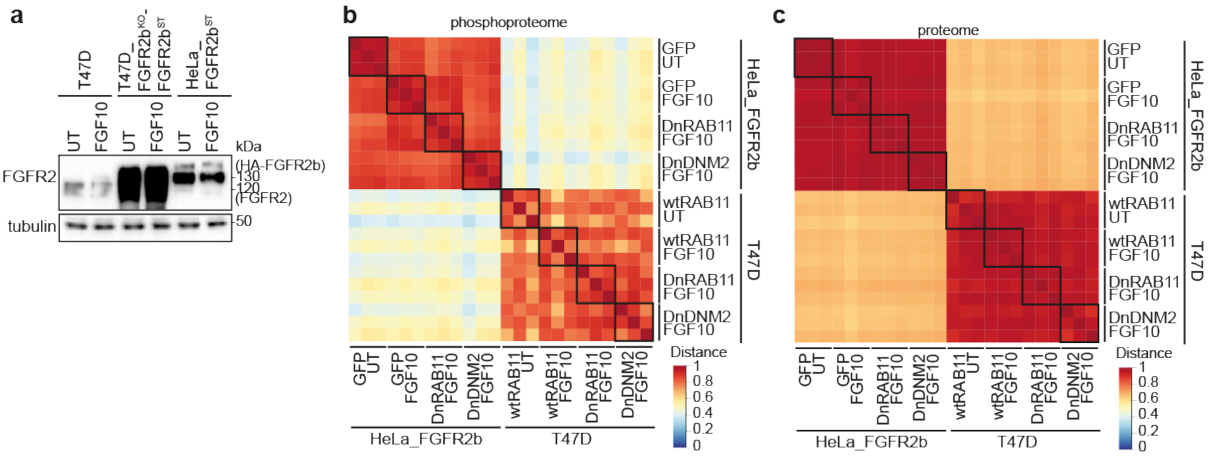

Supplementary Fig. 3. Comparison of the HeLa and T47D proteome and phosphoproteome. a Immunoblot analysis (N>=3 independent biological replicates) with the indicated antibodies of parental T47D, T47D_FGFR2b^{KO}_FGFR2bST and HeLa-FGFR2bST treated with vehicle (UT) or with FGF10 for 40 min. Heat map of the Pearson correlation of the HeLa vs the T47D proteome (**b**) and phosphoproteome (**c**) shows a significant difference between the two epithelial cell lines. Source data are provided as a Source Data file.

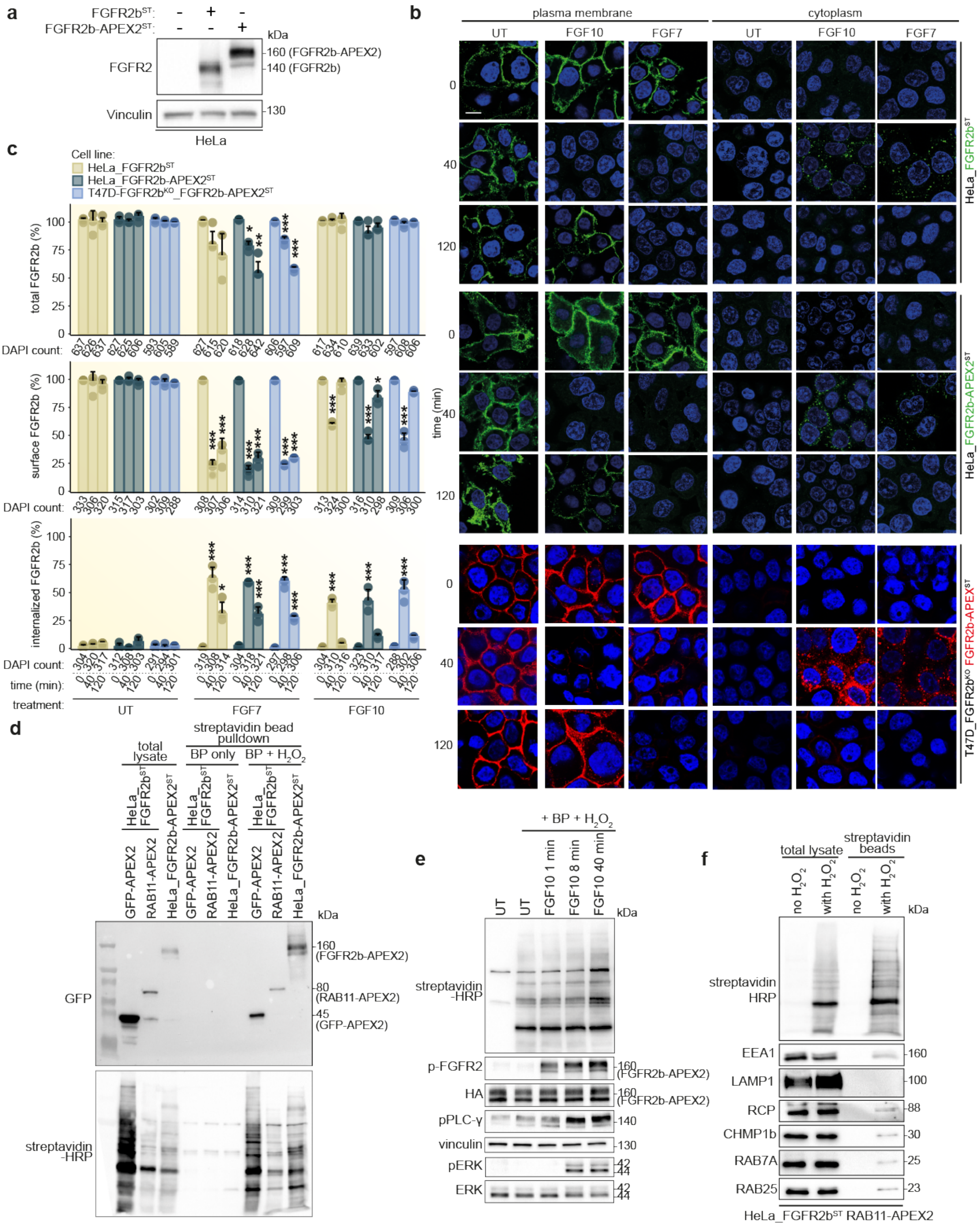

Supplementary Fig. 4 Quality control of the experiments based on APEX2-tagged proteins. a Immunoblot analysis (N>=3 independent biological replicates) with the indicated antibodies of FGFR2b-APEX2 constructs in the indicated cell lines. **b** Representative confocal images of FGFR2b (top panels) and FGFR2b-APEX2 (middle and bottom panels) presence in the cytoplasm and at the plasma membrane in HeLa cells (bottom and middle panels, green) and T47D (bottom panels, red) treated with vehicle (UT) or stimulated with FGF10 or FGF7

for 0, 40, and 120 min. Nuclei are stained with DAPI (blue). Scale bar, 5μm. **c** Quantification of FGFR2b internalization and recycling in the three cell lines, showing the presence (total; top panel), recycled (cell surface; middle panel) and internalized (internalized; bottom panel) FGFR2b upon stimulation. Values represent median ± standard deviation, N>= 3 independent biological replicates where we counted about 100 cells for each condition as indicated below the graph). *p*-value < 0.05 *, *p*-value < 0.005 **, *p*-value < 0.0005 *** (one-way ANOVA, post-hoc Tukey, significance compared to time 0 indicated). **d** Immunoblot analysis (N>=3 independent biological replicates) with the indicated antibodies of HeLa cells transfected with GFP-RAB11-APEX2 or GFP-APEX2 or HA-FGFR2b-APEX2. Total lysates (total) and the pulldown following enrichment of biotinylated samples with streptavidin beads are shown. BP and H_2O_2 treatments show that background biotinylation is negligible. **e** Immunoblot analysis (N>=3 independent biological replicates) with the indicated antibodies of HeLa_FGFR2b-APEX2ST cells treated with vehicle (UT) or with FGF10 for the indicated time points. **f** Immunoblot analysis (N>=3 independent biological replicates) with the indicated antibodies of HeLa_FGFR2bST transfected with RAB11-APEX2 and treated or not with H_2O_2. Source data are provided as a Source Data file.

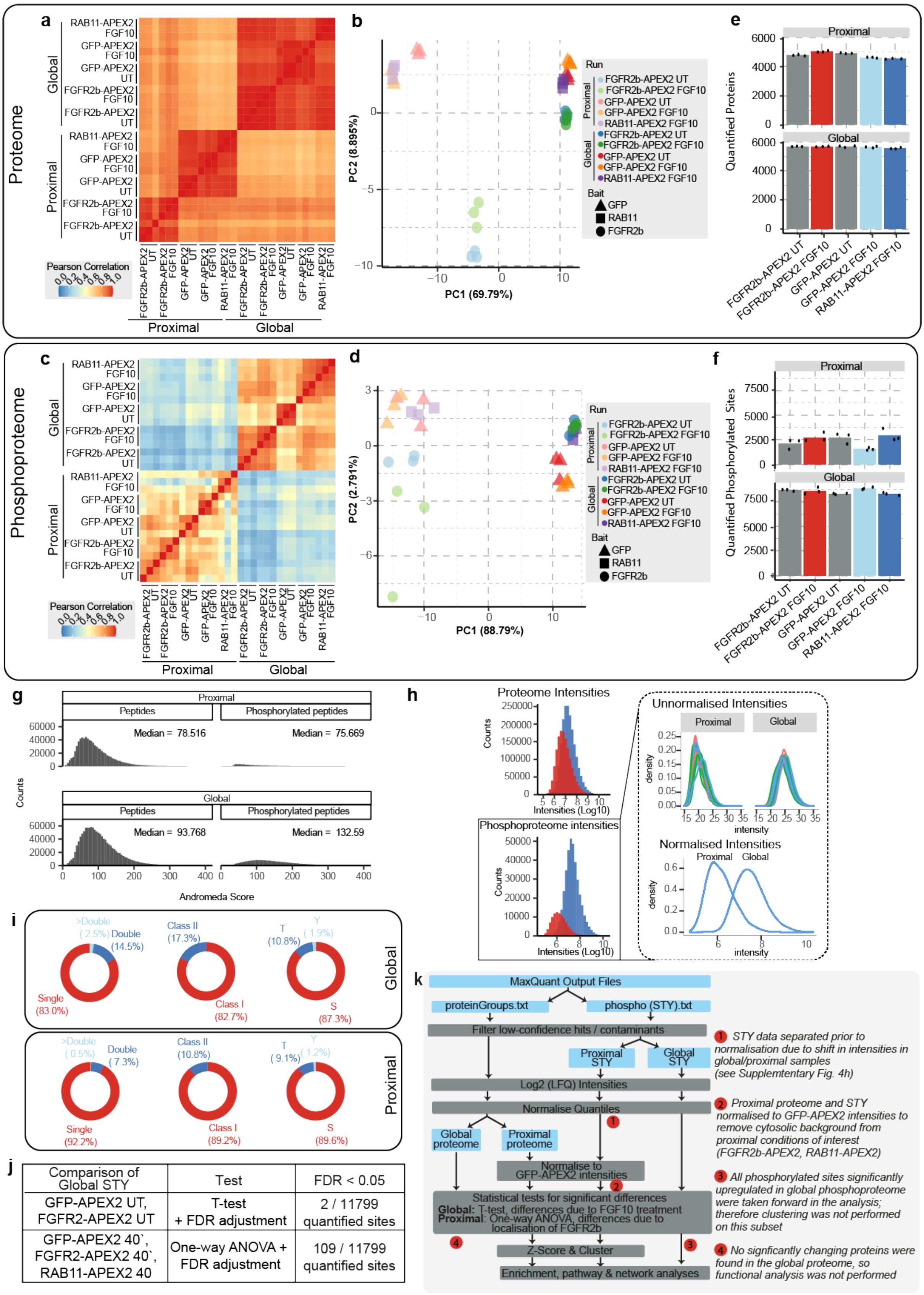

Supplementary Fig. 5. Quality assessment of proximal and global proteomics and phosphoproteomics. a Heat map showing the Pearson correlation of the global and proximal

proteome of HeLa_FGFR2b-APEX2ST wtRAB11, HeLa_FGFR2ST GFP-APEX2 and HeLa_FGFR2ST RAB11-APEX2 treated with FGF10 or with vehicle (UT), showed very good reproducibility among biological replicates (Pearson correlation coefficient higher than or equal to 0.9). Differences were seen among UT and FGF10 stimulated cells expressing different APEX2 constructs in the proximal samples, between UT and FGF10 stimulated cells in the global samples, and between the total and proximal samples more broadly (Pearson correlation coefficient smaller than 0.9). **b** PCA of the proteome samples. **c** Heat map of the Pearson correlation of the phosphoproteome of HeLa_FGFR2b-APEX2ST wtRAB11, HeLa_FGFR2ST GFP-APEX2 and HeLa_FGFR2ST RAB11-APEX2 cells, treated with FGF10 or vehicle (UT) showed very good reproducibility among biological replicates (Pearson correlation coefficient higher than or equal to 0.74). Differences were seen among UT and FGF10 stimulated cells expressing different APEX2 constructs in the proximal samples, between UT and FGF10 stimulated cells in the global samples, and between the total and proximal samples more broadly. **d** PCA of the phosphoproteome samples. **e** Number (n) of protein groups quantified in each condition (Supplementary Data 5). Bars represent mean of N = 3 independent biological replicates. **f** Number (n) of phosphorylated sites quantified in each condition (Supplementary Data 6). Bars represent mean of N = 3 independent biological replicates. **g** The distribution of peptides and phosphorylated peptides Andromeda score showed that most of the peptides were identified with relatively high confidence (median score > 75). **h** Distribution of Log10 intensities of the proteome and phosphoproteome from proximal or global experimental conditions. Inset: densities representing the unnormalized and normalized distributions of intensities. Global and proximal proteome samples were normalized together, whilst phosphoproteome samples were normalized separately. **i** Visualisation of phosphorylated sites containing a single, double of more than double modification (left); percentage of the phosphorylated sites with a localisation probability ≥ 0.75 (Class I) (%) (middle); and of phosphorylated sites with a modification on Serine (S), Threonine (T) and Tyrosine (Y) residues (%) (right). The two panels represent phosphorylated sites quantified in global (top) or proximal. (bottom) experiments. **j** To assess whether expression of APEX2 tags altered the quantification of the phosphoproteome, statistical tests were performed prior to combining the data from the separate mass spectrometry runs (Fig. 3a and Fig. 4a). A t-test was performed between GFP-APEX2 and FGFR2b-APEX2 UT samples, and a one-way ANOVA between GFP-APEX2, FGFR2b-APEX2 and RAB11-APEX2 treated with 100 ng/ml FGF10 for 40 min. P-values were adjusted using the false discovery rate (FDR). In each the UT and FGF10 treated samples, only 2 and 109 phosphorylated sites respectively were found to be statistically different due to the APEX2 tags. Given that this was within the 5% chance of statistical error, we concluded that the APEX2 tags did not affect global phosphoproteome quantification and the global UT and FGF10-treated quantitative values

could be treated as replicates. **k** Data analysis workflow of all quantitative proteomics and phosphoproteomics data. Output files from MaxQuant software were used as input. All data analysis was performed using R and associated packages, as detailed in the methods section.

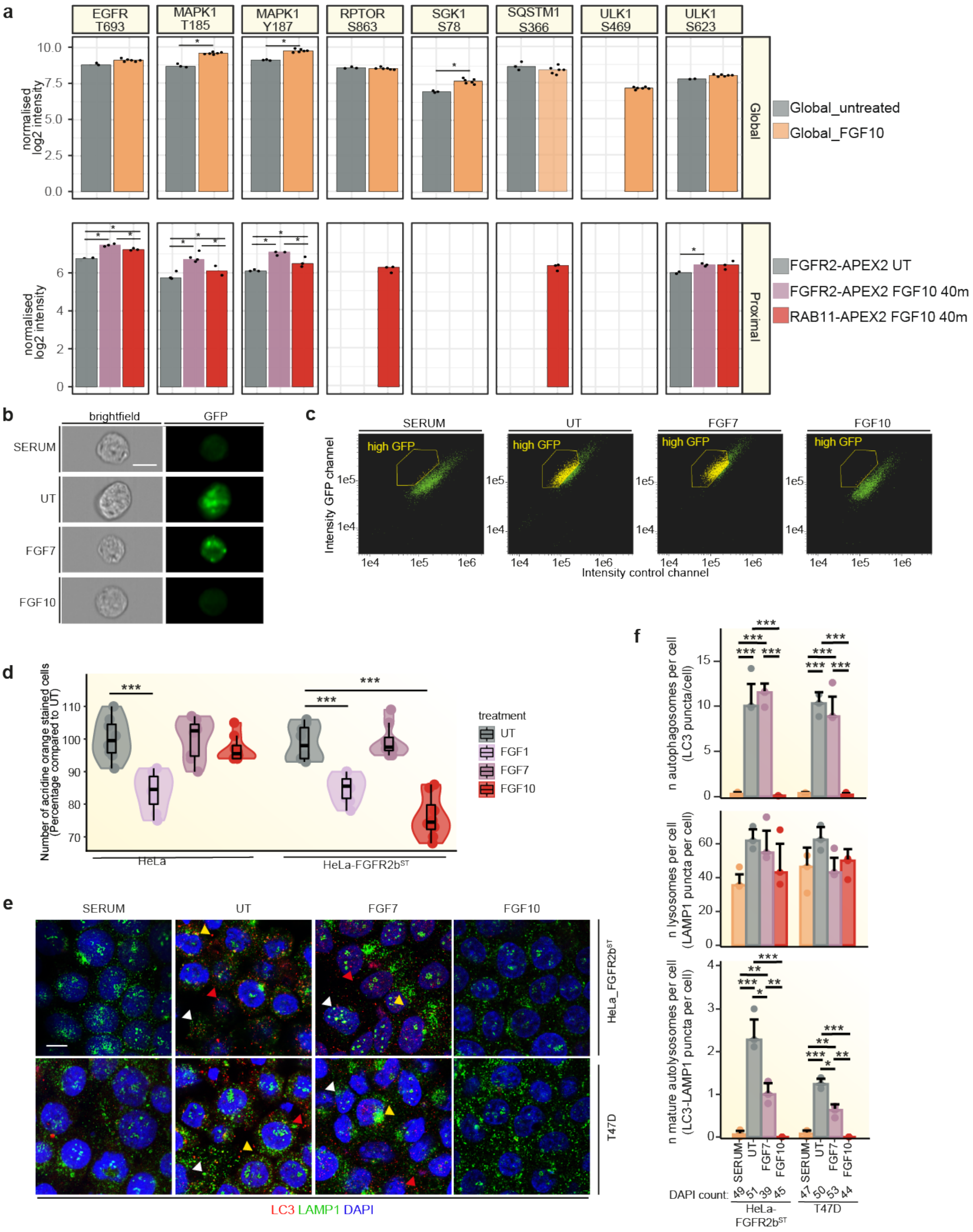

Supplementary Fig 6. FGFR2b signalling and autophagy. a Bar graph showing the log2 intensity of selected phosphorylated sites and proteins from Supplementary Data 6. Global *p*-value < 0.05 * (two-sided permutation t-test with FDR adjustment); proximal *p*-value < 0.05 *, (One-way ANOVA with Tukey). **b** Representative images from Amnis® ImageStream®X Imaging Flow Cytometer analysis of T47D cells grown in serum or starved and treated with vehicle (UT) or with FGF7 or FGF10 for 2h. Scale bar, 10 µm. **c** Gating used in FACS analysis

for quantification of high-GFP, high-autophagy cells shown in b, quantified in Fig. 5h **d** Quantification of autophagy using acridine orange staining and comparing treatment with indicated ligands for 2h to treatment with vehicle (UT). *p*-value < 0.0005 *** (One-way ANOVA with Tukey). N = mean of 6 treated wells examined over 3 independent biological replicates. **e** Representative confocal images of co-localization between LC3 (red) and LAMP1 (green) in HeLa_FGFR2b$_{ST}$ (upper panels) and T47D (lower panels) grown in standard conditions (serum), in starvation medium and treated with vehicle (UT) or in starvation medium followed by stimulation with FGF7 or FGF10 for 2 hours. Scale bar, 30 μm. Red arrowheads indicate LC3-positive vesicles (autophagosomes), white arrowheads indicate LAMP1-positive vesicles (lysosomes), and yellow arrowheads indicate LC3/LAMP1-positive vesicles (mature autolysosomes). **f** Quantification of the number of LC3-, LAMP1- or LC3/LAMP1-positive vesicles per nuclei (indicated below each graph). Values represent median ± SD of N= between 15 and 25 cells counted per image over 3 biological replicates. *p*-value < 0.05 *, < 0.005 **, < 0.0005 *** (One-way ANOVA with Tukey). Source data are provided as a Source Data file.

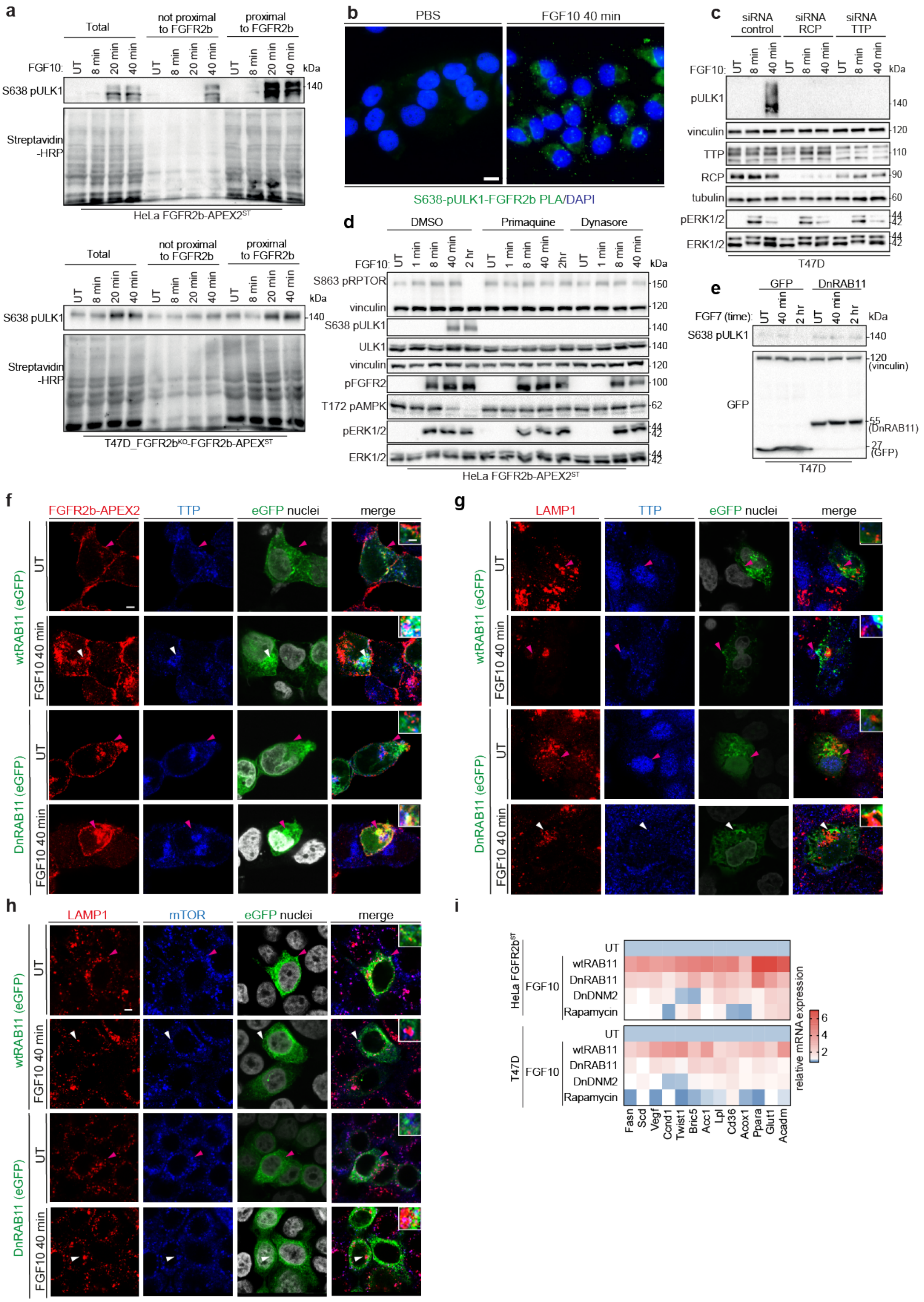

Supplementary Fig. 7. FGFR2b regulates mTOR and ULK1 signalling from the recycling endosomes. a Immunoblot analysis (N>=3 independent biological replicates) with the

indicated antibodies of HeLa FGFR2b-APEX2ST (top) and T47D_FGFR2b^{KO}-FGFR2b-APEX2ST (bottom). Non proximal and proximal samples represent the supernatant and the pulldown following enrichment of biotinylated samples with streptavidin beads, respectively, and run against total lysates (total). UT, treatment with vehicle as control. **b** Representative confocal images corresponding to quantification in Fig. 7c, from Proximity Ligation Assay (PLA) between FGFR2b and S638 pULK1 (green) in T47D cells treated with FGF10 compared to vehicle (UT) Scale bar, 10 μm. **c** Immunoblot analysis (N=3 independent biological replicates) with the indicated antibodies of T47D cells with siRNA-mediated knockdown of TTP or RCP, compared to siRNA control, treated with FGF10 for indicated time points **d** Immunoblot analysis (N=3 independent biological replicates) with the indicated antibodies of HeLa_FGFR2bST cells pre-treated with primaquine or Dynasore for 2 h followed by stimulation with FGF10 for the indicated time points. **e** Immunoblot analysis (N=3 independent biological replicates) with the indicated antibodies of T47D cells transfected with either GFP or DnRAB11 and stimulated with vehicle (UT) or FGF7 for the indicated time points. **f** Representative confocal images of the o-localization between FGFR2b (red) and TTP (blue) in T47D_FGFR2^{KO}_FGFR2b-APEXST transfected with wtRAB11 or DnRAB11 (green) and stimulated with vehicle (UT) or FGF10 for 40 min as indicated. Scale bar, 5 µm. Zoomed images of the region indicated by the arrowheads are shown in the inset (scale bar, 50 μm). The white arrowhead indicates co-localization, and the pink arrowheads indicate lack of co-localization. Representative confocal images of the co-localization between LAMP1 (red) and either TTP (blue) (**g**) or mTOR (blue) (**h**) in T47D_FGFR2^{KO}_FGFR2b-APEXST transfected with wtRAB11 or DnRAB11 (green) and treated with vehicle (UT) or with FGF10 for 40 min as indicated. Scale bar, 5 µm. Zoomed images corresponding to the region indicated by the arrowheads are shown in the inset (scale bar, 50 μm). The white arrowhead indicates co-localization, and the pink arrowheads indicate lack of co-localization. N=3 independent biological replicates. **i** Expression of indicated genes in HeLa FGFR2b or T47D transfected with wtRAB11, DnRAB11 or DnDNM2 or pre-incubated with rapamycin for 2 h followed by stimulation with FGF10 for 4 h. qPCR data are presented as heat map from N= 3 independent biological replicates. Source data are provided as a Source Data file.

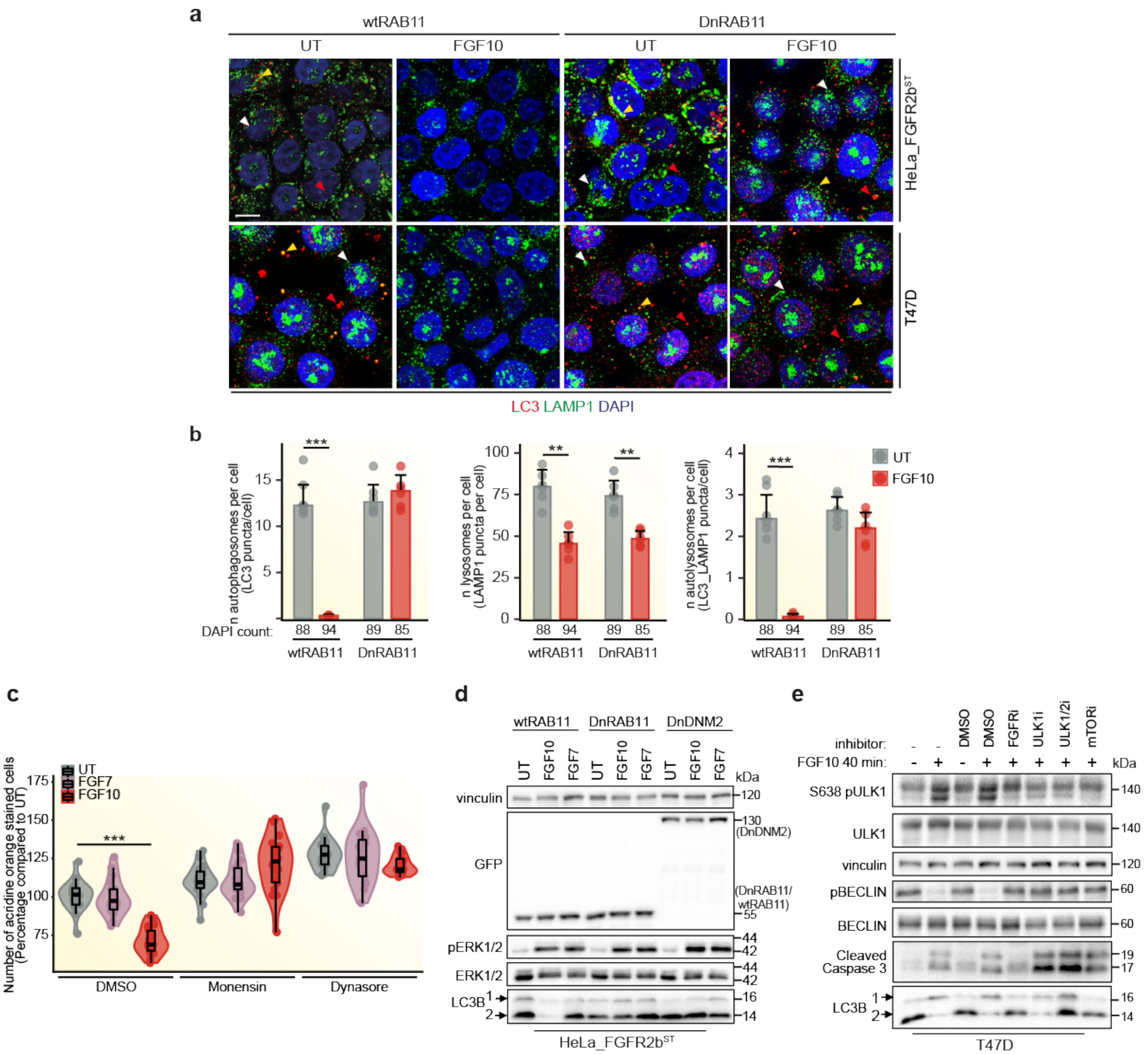

Supplementary Fig. 8. Inhibiting FGFR2b recycling leads to dysregulated autophagy and an altered balance of proliferation and cell death. **a** Representative confocal image of the co-localization between LC3 (red) and LAMP1 (green) in HeLa_FGFR2bST (upper panels) and T47D (lower panels) expressing wtRAB11 or DnRAB11 and stimulated with vehicle (UT) or with FGF10 for 2 hours after starvation. Scale bar, 30 μm. Red arrowheads represent LC3-positive vesicles (autophagosomes), white arrowheads represent LAMP1-positive vesicles (lysosomes), and yellow arrowheads represent LC3/LAMP1-positive vesicles (mature autolysosomes) N=3 independent biological replicates. **b** Quantification of the number of LC3, LAMP1- and LC3/LAMP1-positive vesicles per nuclei. Total number of cells is indicated below each graph. Values represent median ± st dev of N = between 15 and 25 cells per image were counted over 3 independent biological experiments. *P*-value < 0.005 **, *P*-value < 0.0005 *** (one-sided students t-test) **c** Autophagy measured by acridine orange staining after treatment of HeLa_FGFR2bST cells in the indicated conditions. Cells were

incubated with monensin or dynasore for 2 h before incubation with FGF7 or FGF10 for 2 h. N = 3 independent biological replicates where at least 6 treated wells of cells were counted s, *P*-value< 0.0005*** (one-way ANOVA with Tukey test) **d** Immunoblot analysis (N>=3 independent biological replicates) with the indicated antibodies of HeLa_FGFR2bST cells transfected with RAB11 or DnRAB11 or DnDNM2 and stimulated with vehicle (UT), FGF10 or FGF7 for 2 h. **e** Immunoblot analysis (N=3 independent biological replicates) with the indicated antibodies of T47D cells pre-treated for 2 h with FGFR inhibitor (FGFRi) PD173074, ULK1 inhibitor (ULK1i) ULK101, ULK1/2 inhibitor (ULK1/2i) SBI0206965, or mTOR inhibitor (mTORi) Rapamycin and stimulated with FGF10 for 2 h. Source data are provided as a Source Data file.

Supplementary Table 1. List of primers for RT qPCR

Gene	Sequence	Source
ACADM	F-CGTTTTCATTGGAGATCACAGC<br>R-CCAAGACCTCCACAGTTCTCT	In house
ACC1		QuantiTect (#QT00033838)
ACOX1	F-CCCATAAGCCTTTGCCAGGA<br>R-GGCTTCACCTGGGCATACTT	In house
ACOX2	F-GCACCCCGACATAGAGAGC<br>R-CTGCGGAGTGCAGTGTTCT	In house
ACTB	F-GCAAGCAGGAGTATGACGAG<br>R-CAAATAAAGCCATGCCAATC	In house
BIRC5	F-GAGGCTGGCTTCATCCACTG<br>R-CTTTTTGCTTGTTGTTGGTCTCC	In house
CCND1	F-CATCTACACCGACAACTCCATC<br>R-TCTGGCATTTTGGAGAGGAAG	In house
CD36	F-GACCTGCTTATCCAGAAGAC<br>R-TTGCTGCTGTTCATCATAC	In house
FASN	F-CTTCCGAGATTCCATCCTACGC<br>R-TGGCAGTCAGGCTCACAAACG	In house
GLUT1	F-CCAGCTGCCATTGCCGTT<br>R-GACGTAGGGACCACACAGTTGC	In house
LPL	F-GACACAGCTGAGGACACTTG<br>R-TGGAGTCTGGTTCTCTCTTG	In house

PPARA		QuantiTect (#QT01006243)
SCD	F- TCCAGAGGAGGTACTACAAACCT<br><br>R-GCACCACAGCATATCGCAAG	In house
TWIST	F-GTTTTGCAGGCCAGTTTGAT<br><br>R-TGCATGCATTCTCAAGAGGT	In house
VEGF		QuantiTect (#QT01682072)
