# Supplementary material for: Spatially resolved phosphoproteomics reveals fibroblast growth factor receptor recycling-driven regulation of autophagy and survival
Source: Nat Commun. 2022 Nov 3;13:6589. doi: 10.1038/s41467-022-34298-2 (PMC9633600; doi:10.1038/s41467-022-34298-2)
Supplement: Supplementary file 3 — Description os Additional Supplementary Files [file 41467_2022_34298_MOESM3_ESM.docx]

File Name: Supplementary Data 1

Description: HeLa dominant negative normalized, log2 transformed proteome data

File Name: Supplementary Data 2

Description: HeLa dominant negative normalized, log2 transformed, two-sided ANOVA p-value < 0.0001, clustered phosphoproteome data

File Name: Supplementary Data 3

Description: T47D dominant negative normalized, log2 transformed proteome data

File Name: Supplementary Data 4

Description: T47D dominant negative normalized, log2 transformed, clustered phosphoproteome data

File Name: Supplementary Data 5

Description: HeLa APEX2 proteome data

File Name: Supplementary Data 6

Description: HeLa APEX2 phosphoproteome data
