# Supplementary material for: Spatially resolved phosphoproteomics reveals fibroblast growth factor receptor recycling-driven regulation of autophagy and survival
Source: Nat Commun. 2022 Nov 3;13:6589. doi: 10.1038/s41467-022-34298-2 (PMC9633600; doi:10.1038/s41467-022-34298-2)
Supplement: Supplementary file 10 — Reporting Summary [file 41467_2022_34298_MOESM10_ESM.pdf]

nature portfolio

Corresponding author(s): Smith, Francavilla

Last updated by author(s): Sep 21, 2022

Reporting Summary

Nature Portfolio wishes to improve the reproducibility of the work that we publish. This form provides structure for consistency and transparency in reporting. For further information on Nature Portfolio policies, see our Editorial Policies and the Editorial Policy Checklist.

Statistics

For all statistical analyses, confirm that the following items are present in the figure legend, table legend, main text, or Methods section.

n/a	Confirmed	
☐	☒	The exact sample size (*n*) for each experimental group/condition, given as a discrete number and unit of measurement
☐	☒	A statement on whether measurements were taken from distinct samples or whether the same sample was measured repeatedly
☐	☒	The statistical test(s) used AND whether they are one- or two-sided<br>*Only common tests should be described solely by name; describe more complex techniques in the Methods section.*
☐	☒	A description of all covariates tested
☐	☒	A description of any assumptions or corrections, such as tests of normality and adjustment for multiple comparisons
☐	☒	A full description of the statistical parameters including central tendency (e.g. means) or other basic estimates (e.g. regression coefficient) AND variation (e.g. standard deviation) or associated estimates of uncertainty (e.g. confidence intervals)
☐	☒	For null hypothesis testing, the test statistic (e.g. *F*, *t*, *r*) with confidence intervals, effect sizes, degrees of freedom and *P* value noted<br>*Give P values as exact values whenever suitable.*
☐	☒	For Bayesian analysis, information on the choice of priors and Markov chain Monte Carlo settings
☐	☒	For hierarchical and complex designs, identification of the appropriate level for tests and full reporting of outcomes
☐	☒	Estimates of effect sizes (e.g. Cohen's *d*, Pearson's *r*), indicating how they were calculated

Our web collection on statistics for biologists contains articles on many of the points above.

Software and code

Policy information about availability of computer code

Data collection	Raw mass spectrometry data was processed using MaxQuant (version 1.6.2.6 and 1.5.6.5)
Data analysis	All statistical and bioinformatics analyses were done using the freely available software Perseus, version 1.6.5.0 or 1.6.2.1.87, R framework (version 4.2.0) and Bioconductor (release 3.15), Python framework (version 3.7) , SubcellulaRVis (phenome.manchester.ac.uk/subcellular/), STRING (v11.5), Cytoscape (version 3.7.2). Over-representation analysis (ORA) of KEGG terms was performed using Enrichr. R packages, available on CRAN, used were , cluster (version 2.1.4), EnrichR (version 3.1) and imputeLCMD (version 2.1). The Bioconductor package limma (version 3.52.3) was available in release 3.15. Python library statsmodels (version 0.11.1) is available through PyPi and Anaconda.<br>Code used for the analysis of the spatially resolved phosphoproteomics data can be found at: https://github.com/JoWatson2011/APEX2_Analysis_Watson_Ferguson_2022

For manuscripts utilizing custom algorithms or software that are central to the research but not yet described in published literature, software must be made available to editors and reviewers. We strongly encourage code deposition in a community repository (e.g. GitHub). See the Nature Portfolio guidelines for submitting code & software for further information.

Data

Policy information about availability of data

All manuscripts must include a data availability statement. This statement should provide the following information, where applicable:

- Accession codes, unique identifiers, or web links for publicly available datasets
- A description of any restrictions on data availability
- For clinical datasets or third party data, please ensure that the statement adheres to our policy

DATA AVAILABILITY

All data are available in the Source Data files.
The mass spectrometry proteomics data in Thermo Scientific's *.raw format have been deposited to the ProteomeXchange Consortium via the PRIDE 102 partner repository with the following accession codes:
PXD028370 (Signalling and recycling endosome_dataset 01). PXD028330(Signalling and recycling endosome_dataset 02). PXD028371 (Signalling and recycling endosome_dataset 03).
The Uniprot sequence data used in this study are available in the Uniprot database (release 2020-04) under UP000005640 [https://ftp.uniprot.org/pub/databases/uniprot/previous_releases/release-2020_04/knowledgebase/]
The processed mass spectrometry proteomics data generated in this study are provided as Supplementary Data files.
CODE AVAILABILITY
Code has been uploaded on github: https://github.com/JoWatson2011/APEX2_Analysis_Watson_Ferguson_2022 . All other data supporting the findings of this study are available in Source Data.

Field-specific reporting

Please select the one below that is the best fit for your research. If you are not sure, read the appropriate sections before making your selection.

- [x] Life sciences
- [ ] Behavioural & social sciences
- [ ] Ecological, evolutionary & environmental sciences

For a reference copy of the document with all sections, see nature.com/documents/nr-reporting-summary-flat.pdf

Life sciences study design

All studies must disclose on these points even when the disclosure is negative.

Sample size	All the biological experiments have been performed in at least three biological independent replicates based on standard procedure in the field and to verify repeatability and accuracy of results. See for instance Francavilla et al., Mol Cell, 2013 and Smith et al., EMBO J, 2021. Mass Spec-based experiments have been also performed in three biological independent replicates, as is standard for label-free quantification based experiments. See for instance Francavilla et al., Mol Cell, 2013 and Smith et al., EMBO J, 2021. The number of cells to be analyzed in the imaging experiments was chosen to be around 100 or around 10 for the co-localization experiments, following the same procedure as in previously published work. See for instance Francavilla et al., Mol Cell, 2013 and Smith et al., EMBO J, 2021.
Data exclusions	No data were excluded.
Replication	Repeatability and accuracy of results was ensured by performing experiments with three independent biological replicates and validating results with multiple methods (e.g. several assays for autophagy, validating MS data with western blot). Findings were successfully reproduced by more than one person in independent replicates and through different methods.
Randomization	Samples were assigned to different groups based on genetic background (e.g. cells expressing different constructs).
Blinding	Blinding was not relevant considering the multiple number of conditions under examination which made bias analysis extremely unlikely to occur.

Reporting for specific materials, systems and methods

We require information from authors about some types of materials, experimental systems and methods used in many studies. Here, indicate whether each material, system or method listed is relevant to your study. If you are not sure if a list item applies to your research, read the appropriate section before selecting a response.

Materials & experimental systems

n/a	Involved in the study	
[ ]	[x]	Antibodies
[ ]	[x]	Eukaryotic cell lines
[x]	[ ]	Palaeontology and archaeology
[x]	[ ]	Animals and other organisms
[x]	[ ]	Human research participants
[x]	[ ]	Clinical data
[x]	[ ]	Dual use research of concern

Methods

n/a	Involved in the study	
[x]	[ ]	ChIP-seq
[ ]	[x]	Flow cytometry
[x]	[ ]	MRI-based neuroimaging

Antibodies

Antibodies used	Purchased from Cell Signalling Technology: p44/42 MAPK (Erk1/2) (137F5) Rabbit mAb (#4695); FGF Receptor 2 (D4L2V) Rabbit mAb (#23328);GFP (D5.1) Rabbit mAb (#2956); Phospho-FGF Receptor (Tyr653/654) (55H2) Mouse mAb (#3476); Phospho-PLCγ1 (Tyr783) Antibody (rabbit polyclonal) (#2821); PLCγ1 Antibody (rabbit polyclonal) (#2822); Phospho-SHC (Tyr239/240) Antibody (rabbit polyclonal) (#2434S); Shc Antibody (rabbit polyclonal) (#2432); Phospho-FRS2-α (Tyr196) Antibody (rabbit polyclonal) (#3864); EEA1

March 2021

Antibody(rabbit monoclonal) (#3288); LAMP1 (rabbit polyclonal) (#15665); LC3B Antibody (rabbit polyclonal) (#2775); Phospho-Beclin-1 (Ser93) (D9A5G) (rabbit monoclonal) (#14717); Beclin-1 (D40C5) (rabbit monoclonal) (#3495); Phospho-ULK1 (Ser638) (D8K9O) (rabbit monoclonal) (#14205); ULK1 (D8H5) (rabbit monoclonal) (#8054); Raptor (24C12) (rabbit monoclonal) (#2280); Phospho-AMPKα (Thr172) (40H9) (rabbit monoclonal) (#2535); AMPKα (D5A2) (rabbit monoclonal) (#5831); mTOR (7C10) (rabbit monoclonal) (#2983); Rab7 Antibody (rabbit polyclonal) (#2094S); Cleaved Caspase-3 (Asp175) Antibody (rabbit polyclonal) (#9661S)

Purchased from Sigma-Aldrich: Anti-γ-Tubulin (GTU-88) antibody (mouse monoclonal) (#T5326); Anti-Vinculin (hVIN-1) antibody (mouse monocolonal) (#V9264); Anti-HA (12CA5) (mouse monoclonal) (#ROAHA)

Purchased from Abcam: Anti-Histone H3 antibody - Nuclear Marker and ChIP Grade (rabbit polyclonal) (#ab-1791); Anti-Rab25 antibody (rabbit polyclonal) (#ab45855); Anti-LAMP1 – Lyososome Marker (rabbit polyclonal) (#ab24170)

Purchased from Invitrogen: Goat anti-Rabbit IgG (H+L) Secondary Antibody, Alexa Fluor® 488 (Polyclonal) (#A11034); Goat anti-Mouse IgG (H+L) Secondary Antibody, Alexa Fluor® 488 (Polyclonal) (#A11001); Goat anti-Rabbit IgG (H+L) Secondary Antibody, Alexa Fluor® 568 (Polyclonal) (#A11011); Donkey Anti-Mouse IgG (H+L) Secondary Antibody, Alexa Fluor® 647 (Polyclonal) (#A31571); Donkey Anti-Rabbit IgG (H+L) Secondary Antibody, Alexa Fluor® 647 (Polyclonal) (#A31573).

Purchased from other suppliers: Anti-ERK 1/2 Antibody (MK1) (mouse monoclonal) (Santa Cruz Biotechnology, #sc-135900); Anti-FRS2 Antibody (A-5) (mouse monoclonal) (Santa Cruz Biotechnology, #sc-17841); Purified Mouse Anti-EEA1 (14), monoclonal) (BD Bioscience, #610457); LAMP-1/CD107a Lumenal Domain Antibody (mouse polyclonal) (R&D systems, #AF4320); CHMP1b (rabbit polyclonal) (Proteintech, #14639-1-AP); FIP1/RCP Antibody (rabbit polyclonal) (Novus Biologicals, #NBP2-20033); Peroxidase-AffiniPure F(ab')2 Fragment Goat Anti-Mouse IgG (H+L) (Stratech, #115-036-045); Peroxidase-AffiniPure F(ab')2 Fragment Goat Anti-Rabbit IgG (H+L) (Stratech, #115-036-062); anti-LC3 antibody (mouse monoclonal) (MBL, MI86-3)

For immunoblot analysis, γ-tubulin and vinculin were diluted 1:10,000 in 3% BSA (Melford Biolaboratories Ltd), 0.1% tween in PBS. All other primary antibodies were diluted 1:1000 in 3% BSA (Melford Biolaboratories Ltd), 0.1% tween (VWR International Ltd, 8.22184.0500) in PBS. Secondary antibodies: Peroxidase-AffiniPure F(a'')2 Fragment Goat Anti-Mouse IgG (H+L) (Stratech, #115-036-045); Peroxidase-AffiniPure F(a'')2 Fragment Goat Anti-Rabbit IgG (H+L) (Stratech, #115-036-062), were diluted 1:5000 in 3% BSA (Melford Biolaboratories Ltd), 0.1% tween in PBS.

For immunofluorescence, all primary and secondary antibodies were diluted 1:400 in 0.02 % saponin (Sigma), 0.2 % BSA (Melford Biolaboratories Ltd), 0.05 % Tween (VWR International Ltd, 8.22184.0500) in PBS.

Validation

Purchased from Cell Signalling Technology:
1. p44/42 MAPK (Erk1/2) (137F5) Rabbit mAb (#4695): https://www.cellsignal.co.uk/datasheet.jsp?productId=4695&images=1&size=A4
2. FGF Receptor 2 (D4L2V) Rabbit mAb (#23328): https://media.cellsignal.com/s3sds/23328-sds-EGHS-EN-20180201083932000.pdf
3. GFP (D5.1) Rabbit mAb (#2956): https://www.cellsignal.co.uk/datasheet.jsp?productId=2956&images=1&size=A4
4. Phospho-FGF Receptor (Tyr653/654) (55H2) Mouse mAb (#3476): https://media.cellsignal.com/s3sds/3476-sds-EGHS-EN-20180109135602000.pdf
5. Phospho-PLCγ1 (Tyr783) Antibody (rabbit polyclonal) (#2821): https://media.cellsignal.com/s3sds/2821-sds-EGHS-EN-20180214130908000.pdf
6. PLCγ1 Antibody (rabbit polyclonal) (#2822): https://www.cellsignal.co.uk/datasheet.jsp?productId=2822&images=1&size=A4
7. Phospho-SHC (Tyr239/240) Antibody (rabbit polyclonal) (#2434S): https://media.cellsignal.com/s3sds/2434-sds-EGHS-EN-20180212151122000.pdf
8. Shc Antibody (rabbit polyclonal) (#2432): https://media.cellsignal.com/s3sds/2432-sds-EGHS-EN-20180212151030000.pdf
9. Phospho-FRS2-α (Tyr196) Antibody (rabbit polyclonal) (#3864)
10. EEA1 Antibody(rabbit monoclonal) (#3288): https://media.cellsignal.com/s3sds/3288-sds-EGHS-EN-20180105150024000.pdf
11. LAMP1 (rabbit polyclonal) (#15665): https://media.cellsignal.com/s3sds/15665-sds-EGHS-EN-20180201090510000.pdf
12. LC3B Antibody (rabbit polyclonal) (#2775): https://www.cellsignal.co.uk/datasheet.jsp?productId=2775&images=1&size=A4
13. Phospho-Beclin-1 (Ser93) (D9A5G) (rabbit monoclonal) (#14717): https://www.cellsignal.co.uk/datasheet.jsp?productId=14717&images=1&size=A4
14. Beclin-1 (D40C5) (rabbit monoclonal) (#3495): https://www.cellsignal.co.uk/datasheet.jsp?productId=3495&images=1&size=A4
15. Phospho-ULK1 (Ser638) (D8K9O) (rabbit monoclonal) (#14205): https://www.cellsignal.co.uk/datasheet.jsp?productId=14205&images=1&size=A4
16. ULK1 (D8H5) (rabbit monoclonal) (#8054): https://www.cellsignal.co.uk/datasheet.jsp?productId=8054&images=1&size=A4
17. Raptor (24C12) (rabbit monoclonal) (#2280): https://www.cellsignal.co.uk/datasheet.jsp?productId=2280&images=1&size=A4
18. Phospho-AMPKα (Thr172) (40H9) (rabbit monoclonal) (#2535): https://www.cellsignal.co.uk/datasheet.jsp?productId=2535&images=1&size=A4
19. AMPKα (D5A2) (rabbit monoclonal) (#5831): https://www.cellsignal.co.uk/datasheet.jsp?productId=5831&images=1&size=A4
20. mTOR (7C10) (rabbit monoclonal) (#2983): https://www.cellsignal.co.uk/datasheet.jsp?productId=2983&images=1&size=A4
21. Rab7 Antibody (rabbit polyclonal) (#2094S): https://www.cellsignal.co.uk/datasheet.jsp?productId=2094&images=1&size=A4
22. Cleaved Caspase-3 (Asp175) Antibody (rabbit polyclonal) (#9661S): https://www.cellsignal.co.uk/datasheet.jsp?productId=9661&images=1&size=A4

Purchased from Sigma-Aldrich:
1. Anti-γ-Tubulin (GTU-88) antibody (mouse monoclonal) (#T5326): https://www.sigmaaldrich.com/deepweb/assets/sigmaaldrich/product/documents/331/901/t5326pis-mk.pdf
2. Anti-Vinculin (hVIN-1) antibody (mouse monocolonal) (#V9264): https://www.sigmaaldrich.com/deepweb/assets/sigmaaldrich/product/documents/352/187/v9264dat.pdf

3. Anti-HA (12CA5) (mouse monoclonal) (#ROAHA): https://www.sigmaaldrich.com/deepweb/assets/sigmaaldrich/product/documents/237/191/roahabul.pdf

Purchased from Abcam:
1. Anti-Histone H3 antibody - Nuclear Marker and ChIP Grade (rabbit polyclonal) (#ab-1791): https://www.abcam.com/histone-h3-antibody-nuclear-marker-and-chip-grade-ab1791.pdf
2. Anti-Rab25 antibody (rabbit polyclonal) (#ab45855): https://www.abcam.com/rab25-antibody-ab45855.pdf
3. Anti-LAMP1 – Lyososome Marker (rabbit polyclonal) (#ab24170): https://www.abcam.com/lamp1-antibody-lysosome-marker-ab24170.pdf

Purchased from Invitrogen:
1. Goat anti-Rabbit IgG (H+L) Secondary Antibody, Alexa Fluor® 488 (Polyclonal) (#A11034): https://www.thermofisher.com/order/genome-database/dataSheetPdf?producttype=antibody&productsubtype=antibody_secondary&productId=A-11034&version=251
2. Goat anti-Mouse IgG (H+L) Secondary Antibody, Alexa Fluor® 488 (Polyclonal) (#A11001): https://www.thermofisher.com/order/genome-database/dataSheetPdf?producttype=antibody&productsubtype=antibody_secondary&productId=A-11001&version=251
3. Goat anti-Rabbit IgG (H+L) Secondary Antibody, Alexa Fluor® 568 (Polyclonal) (#A11011): https://www.thermofisher.com/order/genome-database/dataSheetPdf?producttype=antibody&productsubtype=antibody_secondary&productId=A-11011&version=251
4. Donkey Anti-Mouse IgG (H+L) Secondary Antibody, Alexa Fluor® 647 (Polyclonal) (#A31571): https://www.thermofisher.com/order/genome-database/dataSheetPdf?producttype=antibody&productsubtype=antibody_secondary&productId=A-31571&version=251
5. Donkey Anti-Rabbit IgG (H+L) Secondary Antibody, Alexa Fluor® 647 (Polyclonal) (#A31573): https://www.thermofisher.com/order/genome-database/dataSheetPdf?producttype=antibody&productsubtype=antibody_secondary&productId=A-31573&version=251
purchased from other suppliers:
1. Anti-ERK 1/2 Antibody (MK1) (mouse monoclonal) (Santa Cruz Biotechnology, #sc-135900): https://datasheets.scbt.com/sc-135900.pdf
2. Anti-FRS2 Antibody (A-5) (mouse monoclonal) (Santa Cruz Biotechnology, #sc-17841): https://datasheets.scbt.com/sc-17841.pdf
3. Purified Mouse Anti-EEA1 (14), monoclonal) (BD Bioscience, #610457): https://www.bdbiosciences.com/content/bdb/paths/generate-tds-document.gb.610457.pdf
4. LAMP-1/CD107a Lumenal Domain Antibody (mouse polyclonal) (R&D systems, #AF4320): https://resources.rndsystems.com/pdfs/datasheets/af4320.pdf?v=20220923
5. CHMP1b (rabbit polyclonal) (Proteintech, #14639-1-AP) https://www.ptglab.com/products/pictures/pdf/14639-1-AP.pdf
6. FIP1/RCP Antibody (rabbit polyclonal) (Novus Biologicals, #NBP2-20033) https://www.novusbio.com/PDFs/NBP2-20033.pdf
7. Peroxidase-AffiniPure F(ab')2 Fragment Goat Anti-Mouse IgG (H+L) (Stratech, #115-036-045): https://www.stratech.co.uk/products/111-036-045-JIR/
8. Peroxidase-AffiniPure F(ab')2 Fragment Goat Anti-Rabbit IgG (H+L) (Stratech, #115-036-062): https://www.stratech.co.uk/products/115-036-062-JIR/
9. mouse anti-LC3 antibody (MBL, MI86-3): https://www.mblintl.com/products/m186-3/

Eukaryotic cell lines

Policy information about cell lines

Cell line source(s)	Human epithelial cell lines were purchased from ATCC: HeLa (CCL-2), T47D (HTB-133), BT20 (HTB-19).
Authentication	Cell lines were authenticated in 2019 through short tandem repeat (STA) analysis of 21 markers by Eurofins Genomics.
Mycoplasma contamination	Cell lines were checked monthly for mycoplasma via a PCR-based detection assay (Venor®GeM – Cambio). All cell lines tested negative.
Commonly misidentified lines (See ICLAC register)	We did not used any.

Flow Cytometry

Plots

Confirm that:

- [x] The axis labels state the marker and fluorochrome used (e.g. CD4-FITC).
- [x] The axis scales are clearly visible. Include numbers along axes only for bottom left plot of group (a 'group' is an analysis of identical markers).
- [x] All plots are contour plots with outliers or pseudocolor plots.
- [x] A numerical value for number of cells or percentage (with statistics) is provided.

Methodology

Sample preparation	Human breast cancer cell line, T47D, was treated with FGF7, FGF10 or PBS for 2 hours then trypsinised and pelleted. Cell pellets were then stained using a CYTO-ID staining solution and fixed in 4% formaldehyde.

Instrument	Imaging cytometry data was collected on a 4-laser, dual camera Amnis ImageStreamX MkII (ISX). Brightfield, CYTO-ID and scatter channels were collected using channels 1, 2 and 6 respectively within the INSPIRE software. Brightfield images were illuminated with onboard LEDs, CYTO-ID was excited with a 488nm laser set at 100mW and scatter was detected as cells passed through a 785nm laser set to 3.75mW. CYTO-ID emission was collected through a bandpass filter 480-560nm. All other channels were deactivated in order to reduce file size.
Software	Data was acquired from the ISX with INSPIRE software, version 201.1 (Amnis Corp.).<br>Data analysis of acquired data was performed in IDEAS software, version 6.2 (Amnis Corp.) where raw data (*.rif) files were background corrected and converted to data analysis files (*.daf) for gating and image analysis.
Cell population abundance	Scatter plots displaying the 'area' and 'aspect ratio' of cells as they were imaged were used to identify single cell events over doublet and aggregated cells. Single cells were then further segregated into in-focus cells based on the 'Gradient RMS' parameter of the brightfield channel in the INSPIRE software. Five thousand in-focus, single cells were collected for each data set. Single, in-focus cells were approximately 85% of the total cell events run on the ISX.
Gating strategy	Analysis was performed in the IDEAS software package. Scatter plots were gated to ensure the removal of any doublet events (<2% of the data set) and resultant data was plotted for the intensity of the CYTO-ID signal against the intensity of the scatter channel. An polygonal analysis gate was hand drawn to locate CYTO-ID positive events, with the use of the unlabelled controls as a guide. Data was exported from the IDEAS software as csv files and analysed in Excel.

☒ Tick this box to confirm that a figure exemplifying the gating strategy is provided in the Supplementary Information.
