# Supplementary material for: Spatially resolved phosphoproteomics reveals fibroblast growth factor receptor recycling-driven regulation of autophagy and survival
Source: Nat Commun. 2022 Nov 3;13:6589. doi: 10.1038/s41467-022-34298-2 (PMC9633600; doi:10.1038/s41467-022-34298-2)

Figure 1c

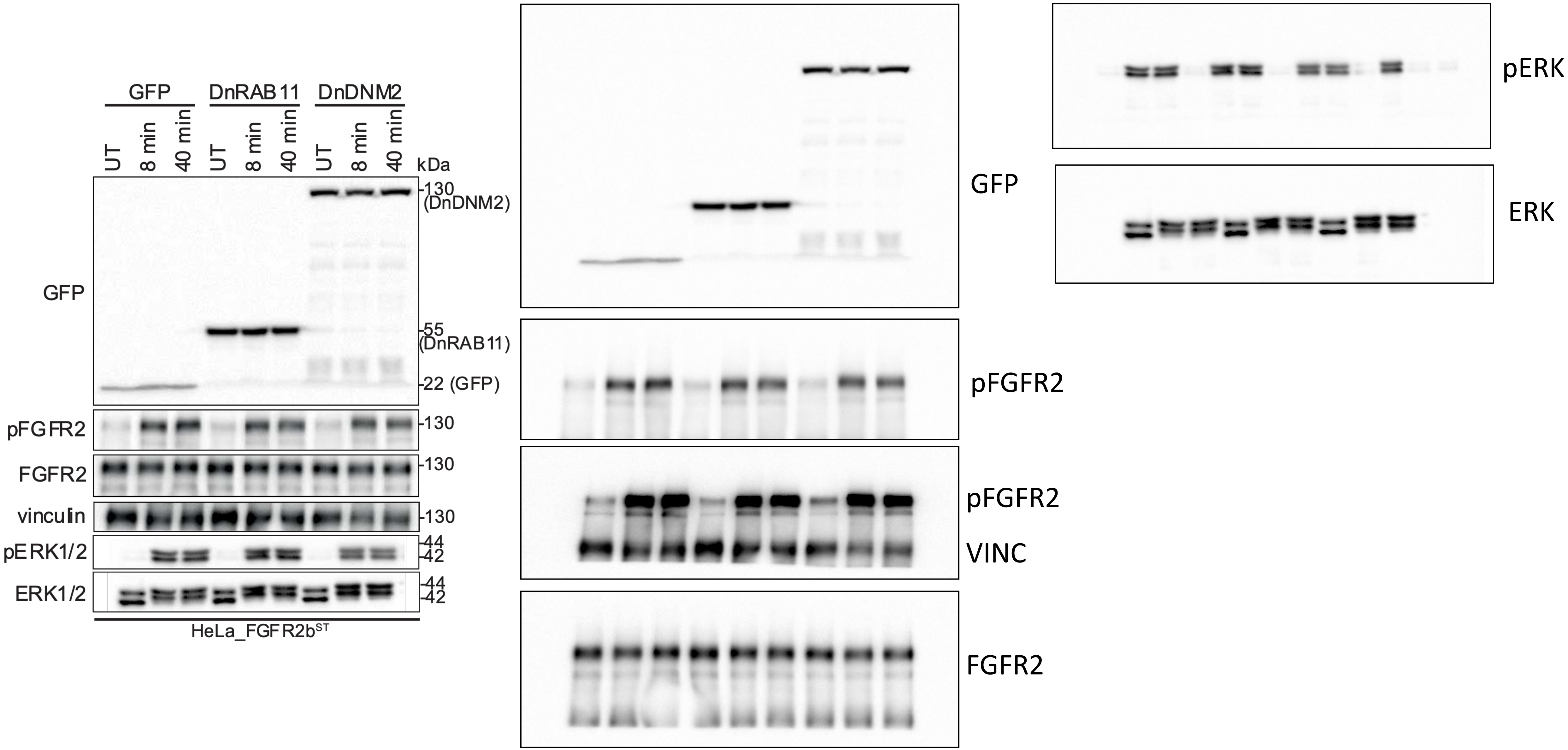
GFP
DnRAB11
DnDNM2
UT
8 min
40 min
kDa
GFP
-130 (DnDNM2)
-55 (DnRAB11)
-22 (GFP)
pFGFR2
-130
FGFR2
-130
vinculin
-130
pERK1/2
-44
-42
ERK1/2
-44
-42
HeLa_FGFR2b^ST
GFP
pFGFR2
pFGFR2
VINC
FGFR2
pERK
ERK

Figure 3b

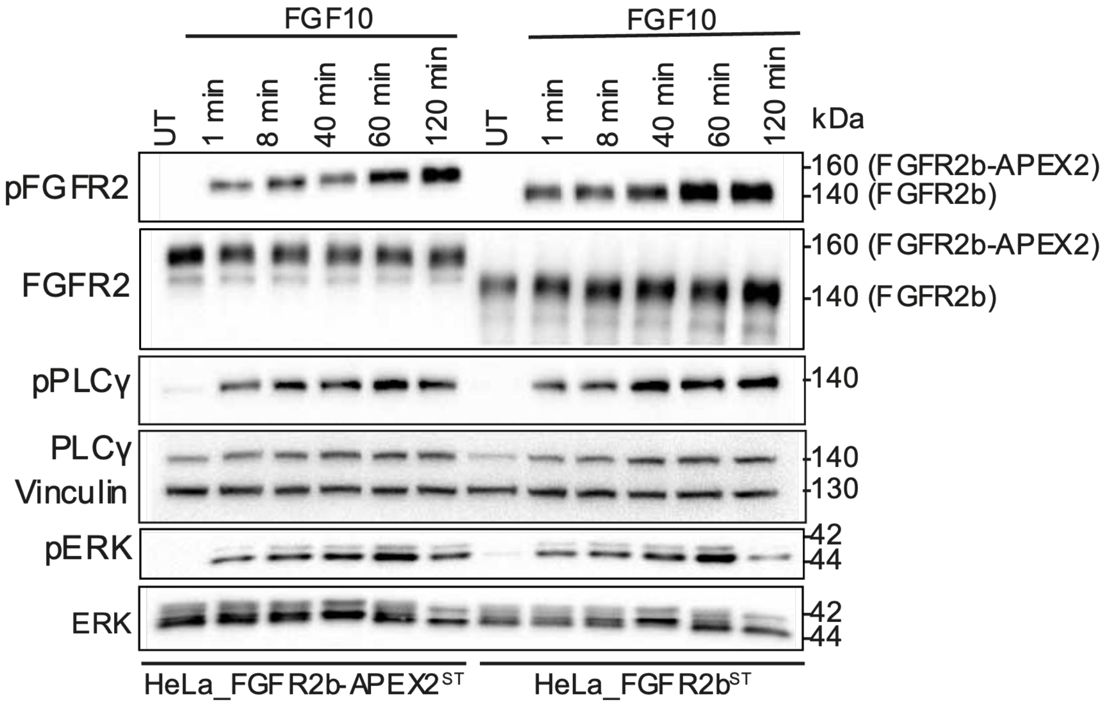

FGF10
FGF10
UT
1 min
8 min
40 min
60 min
120 min
UT
1 min
8 min
40 min
60 min
120 min
kDa
pFGFR2
160 (FGFR2b-APEX2)
140 (FGFR2b)
FGFR2
160 (FGFR2b-APEX2)
140 (FGFR2b)
pPLCγ
140
PLCγ
Vinculin
140
130
pERK
42
44
ERK
42
44
HeLa_FGFR2b-APEX2ST
HeLa_FGFR2bST

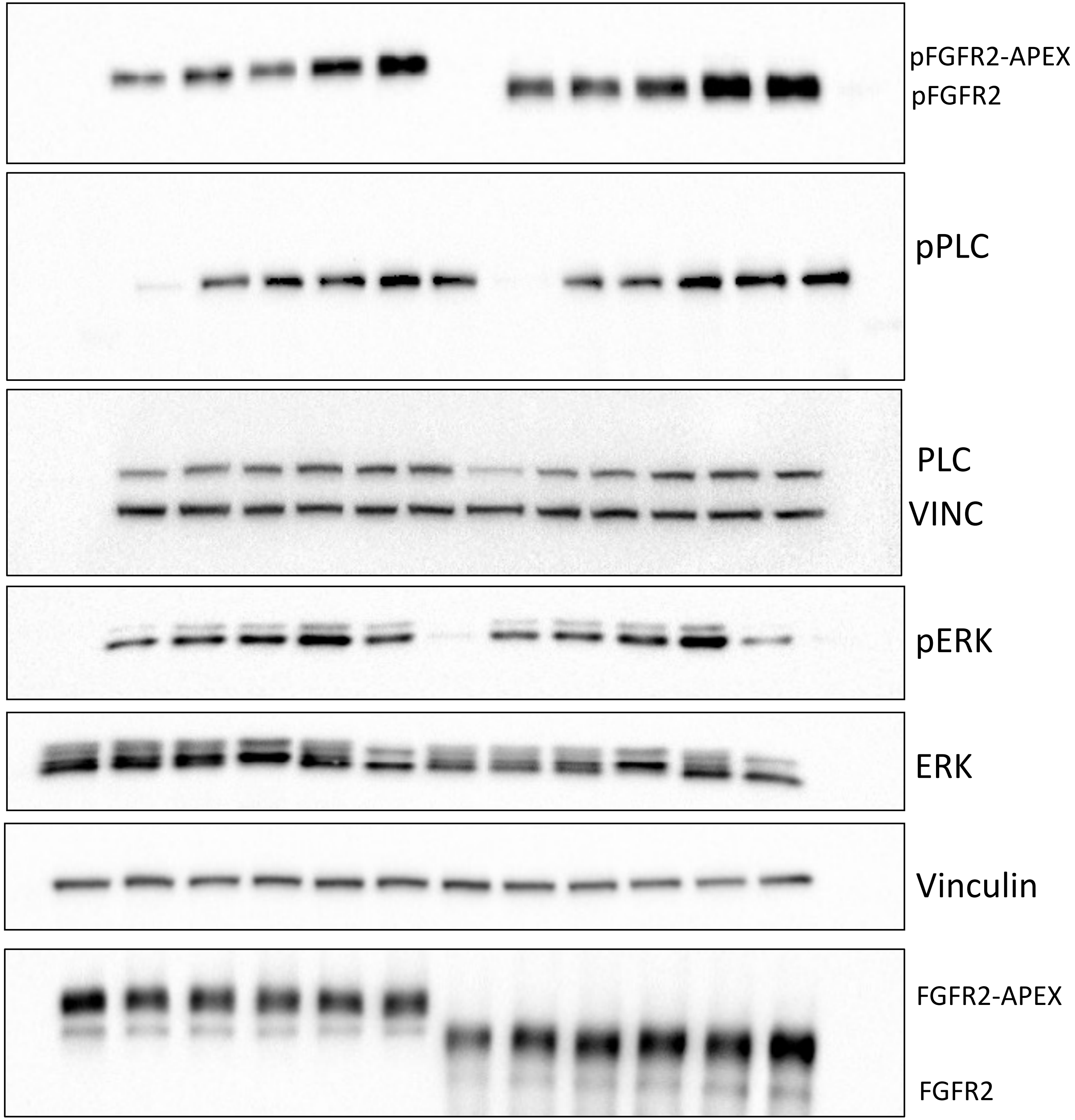

pFGFR2-APEX
pFGFR2
pPLC
PLC
VINC
pERK
ERK
Vinculin
FGFR2-APEX
FGFR2

Figure 3e

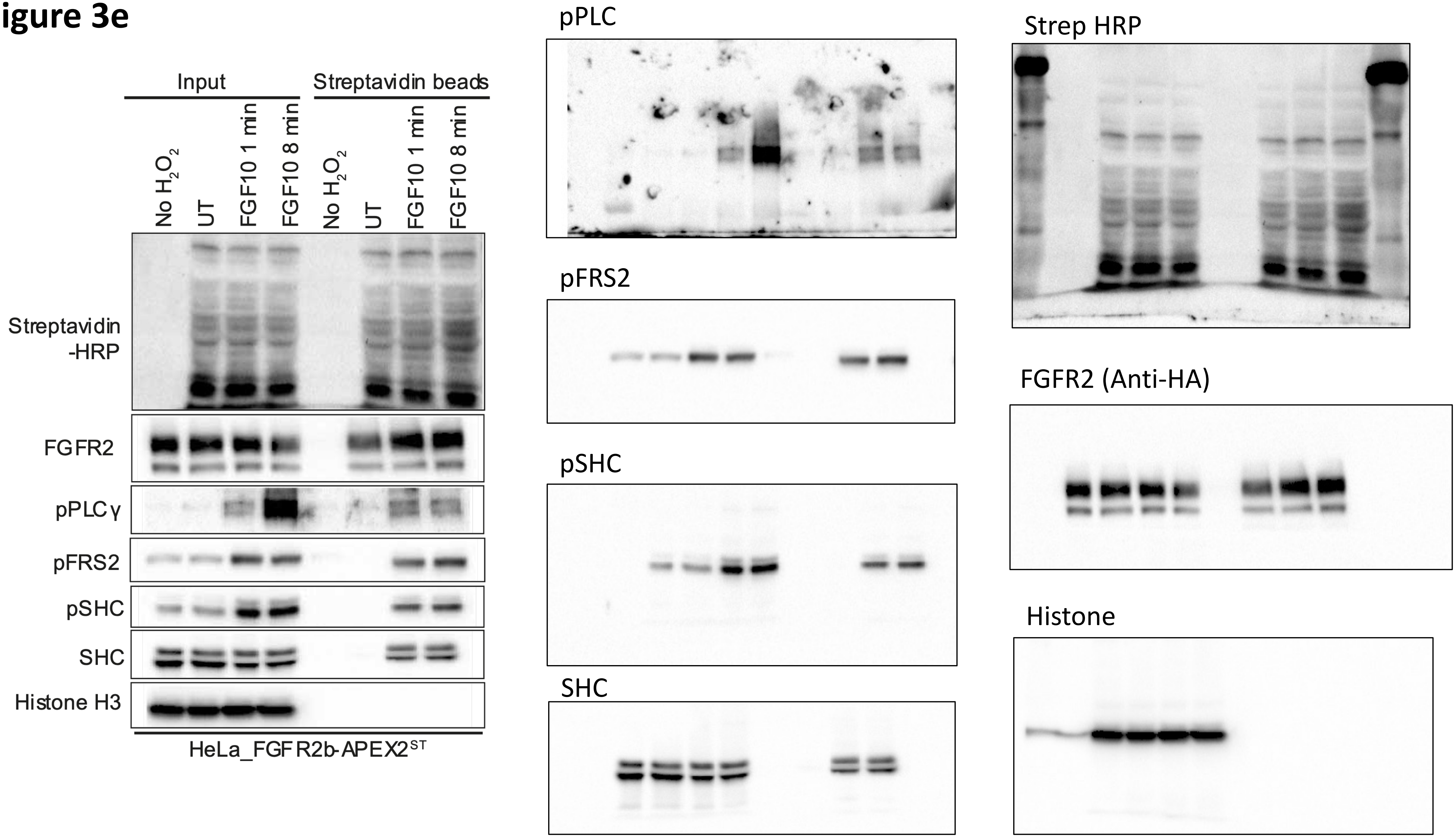
Input
Streptavidin beads
No H_2O_2
UT
FGF10 1 min
FGF10 8 min
No H_2O_2
UT
FGF10 1 min
FGF10 8 min
Streptavidin -HRP
FGFR2
pPLCγ
pFRS2
pSHC
SHC
Histone H3
HeLa_FGFR2b-APEX2ST
pPLC
pFRS2
pSHC
SHC
Strep HRP
FGFR2 (Anti-HA)
Histone

Figure 3g

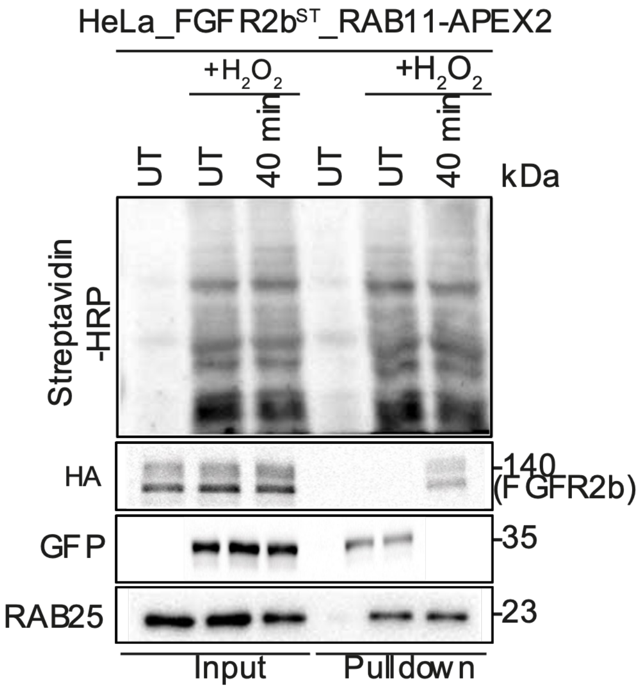
HeLa_FGFR2b^ST_RAB11-APEX2
+H2O2
+H2O2
UT
UT
40 min
UT
UT
40 min
kDa
Streptavidin
-HRP
HA
-140
(FGFR2b)
GFP
-35
RAB25
-23
Input
Pulldown

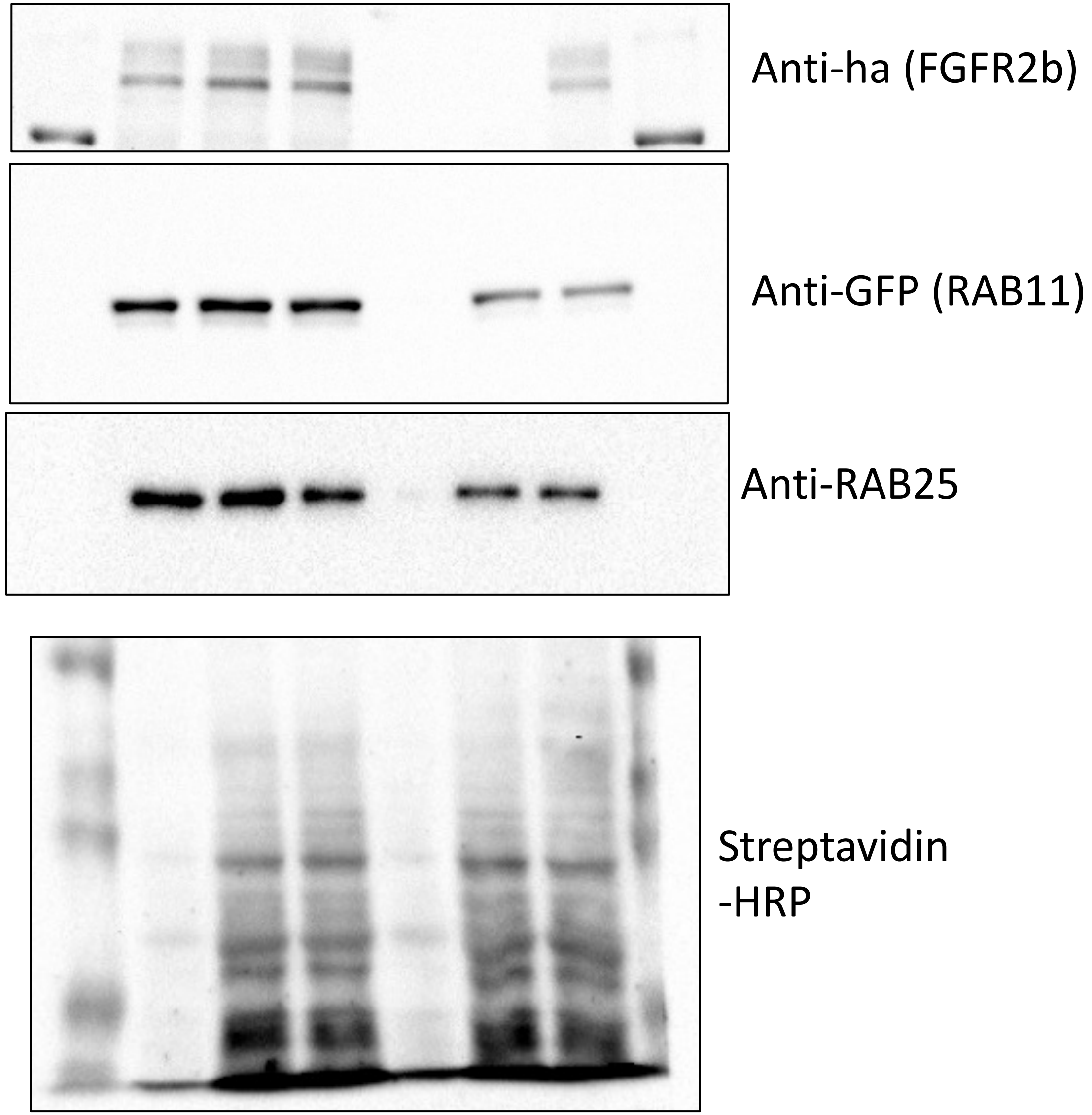
Anti-ha (FGFR2b)
Anti-GFP (RAB11)
Anti-RAB25
Streptavidin
-HRP

Figure 5b

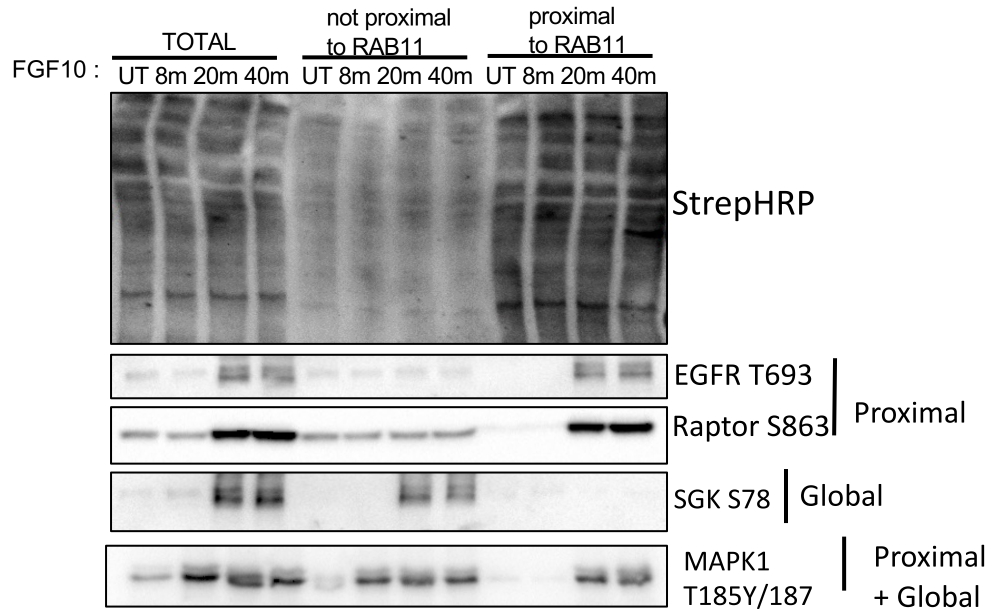
TOTAL
not proximal to RAB11
proximal to RAB11
FGF10 :
UT 8m 20m 40m UT 8m 20m 40m UT 8m 20m 40m
StrepHRP
EGFR T693
Raptor S863
Proximal
SGK S78
Global
MAPK1 T185Y/187
Proximal + Global

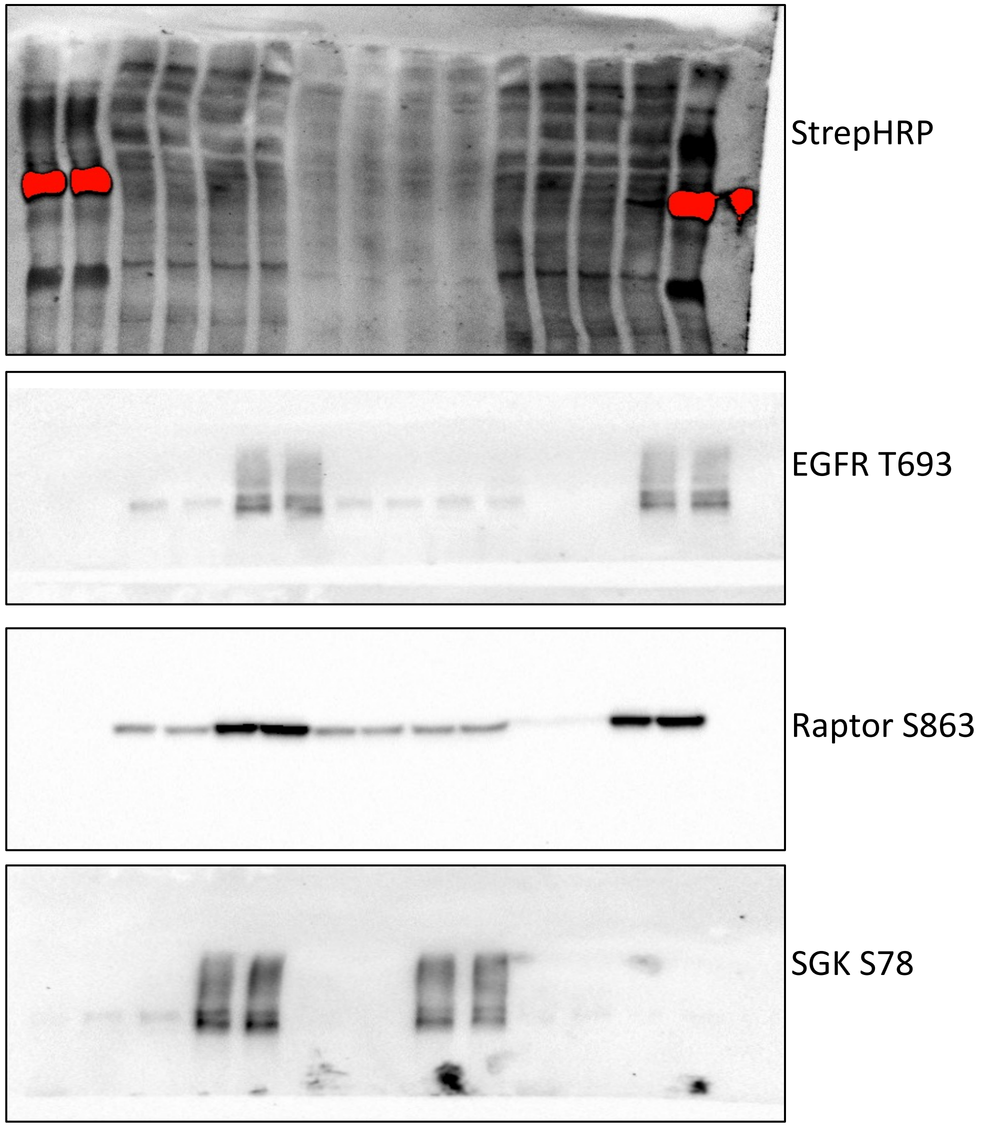
StrepHRP
EGFR T693
Raptor S863
SGK S78

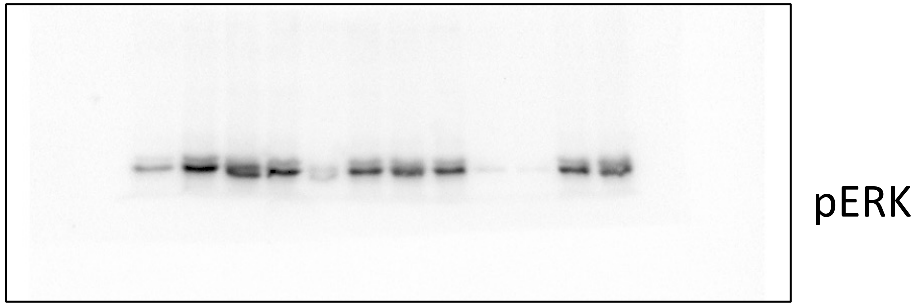
pERK

Figure 5e

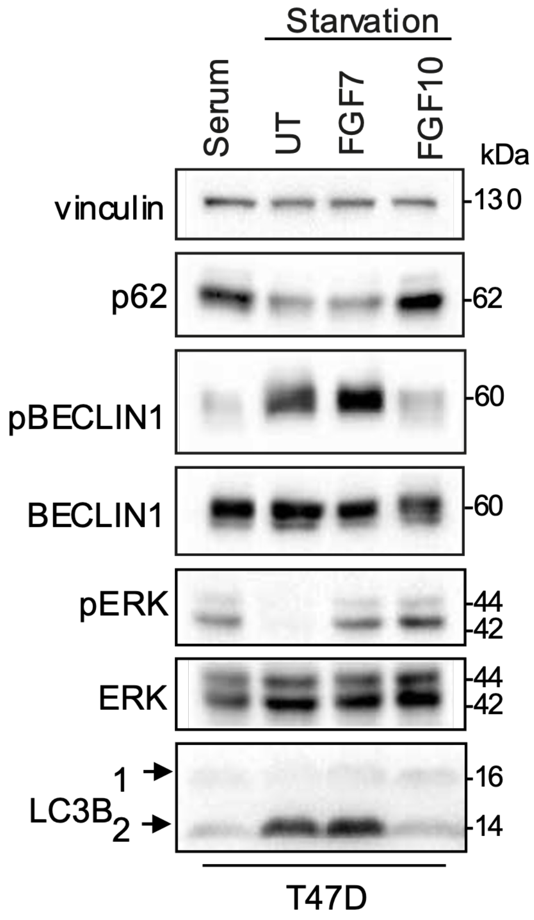

Starvation
Serum
UT
FGF7
FGF10
kDa
vinculin
130
p62
62
pBECLIN1
60
BECLIN1
60
pERK
44
42
ERK
44
42
1
LC3B
2
16
14
T47D

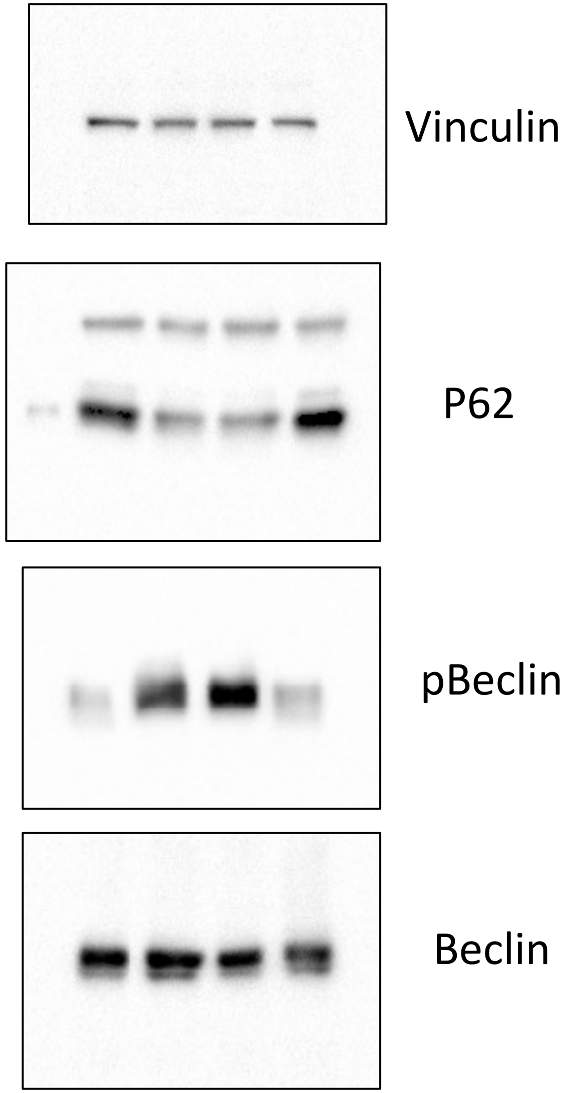

Vinculin
P62
pBeclin
Beclin

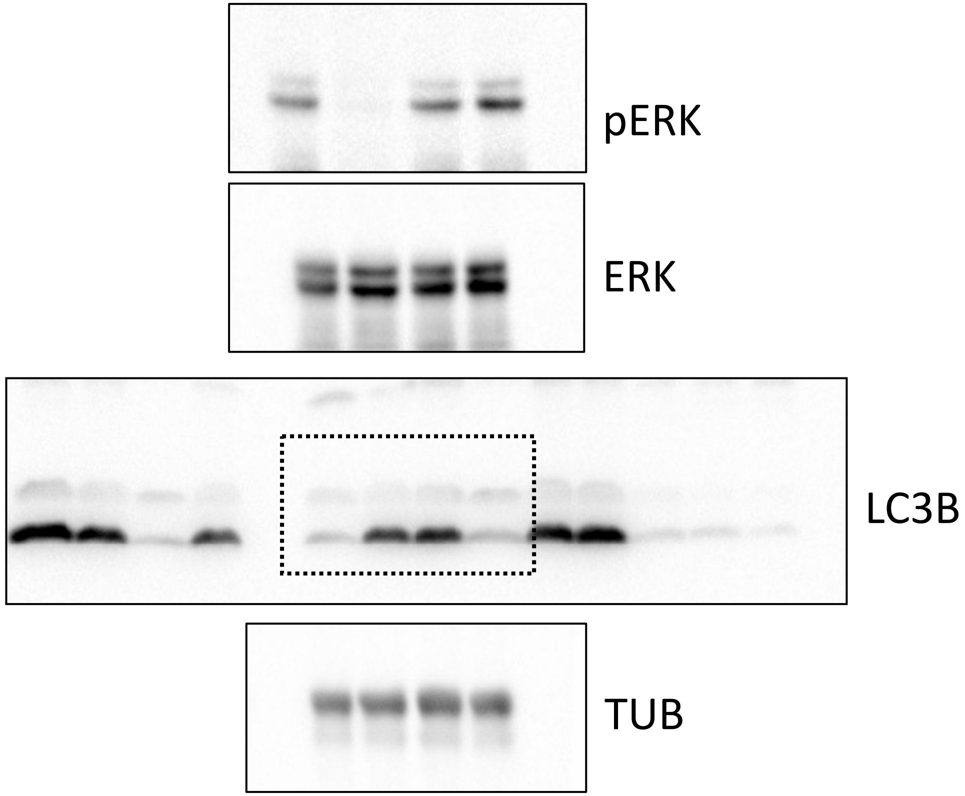

pERK
ERK
LC3B
TUB

Figure 5f

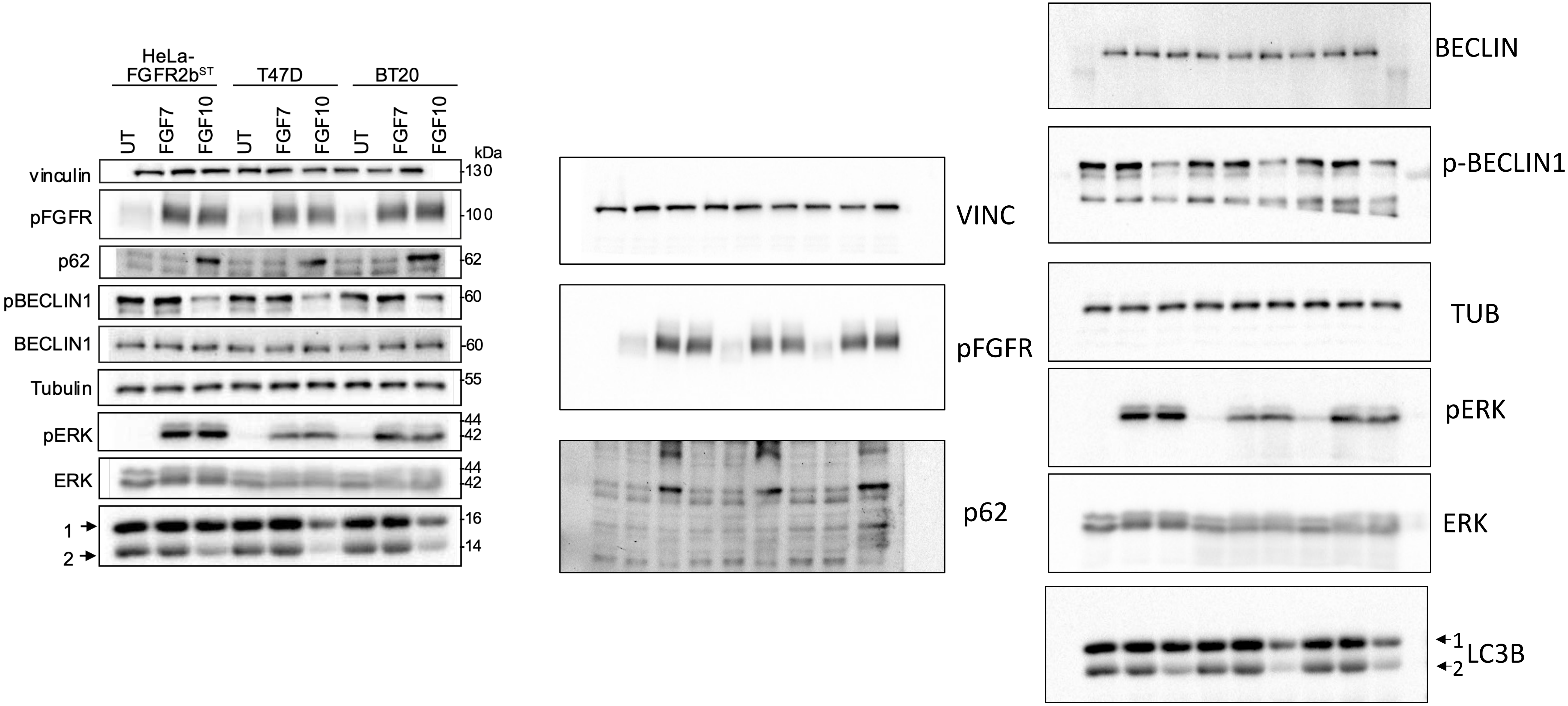
HeLa-FGFR2b^ST
T47D
BT20
UT
FGF7
FGF10
UT
FGF7
FGF10
UT
FGF7
FGF10
kDa
vinculin
130
pFGFR
100
p62
62
pBECLIN1
60
BECLIN1
60
Tubulin
55
pERK
44
42
ERK
44
42
1
2
16
14
VINC
pFGFR
p62
BECLIN
p-BECLIN1
TUB
pERK
ERK
1
2
LC3B

Figure 6b

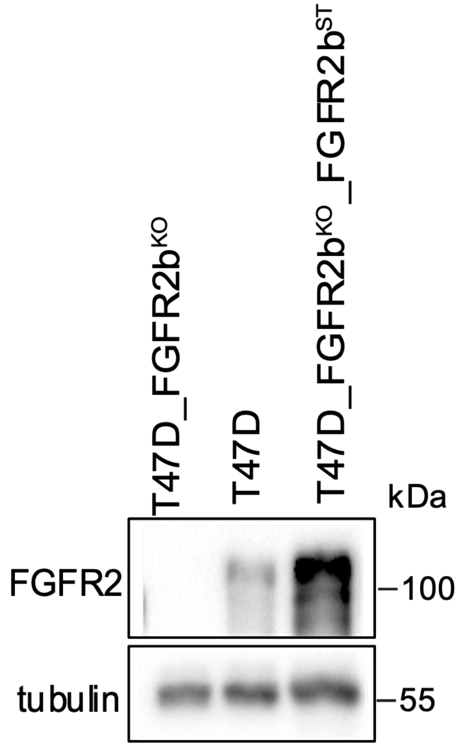

T47D_FGFR2b^KO
T47D
T47D_FGFR2b^KO_FGFR2b^ST
kDa
FGFR2
100
tubulin
55

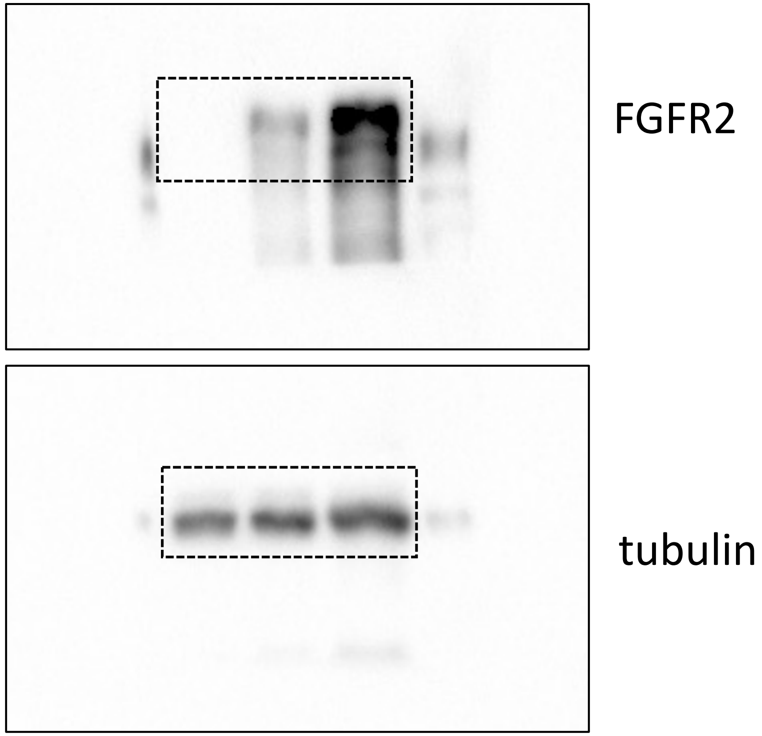

FGFR2
tubulin

Figure 6d

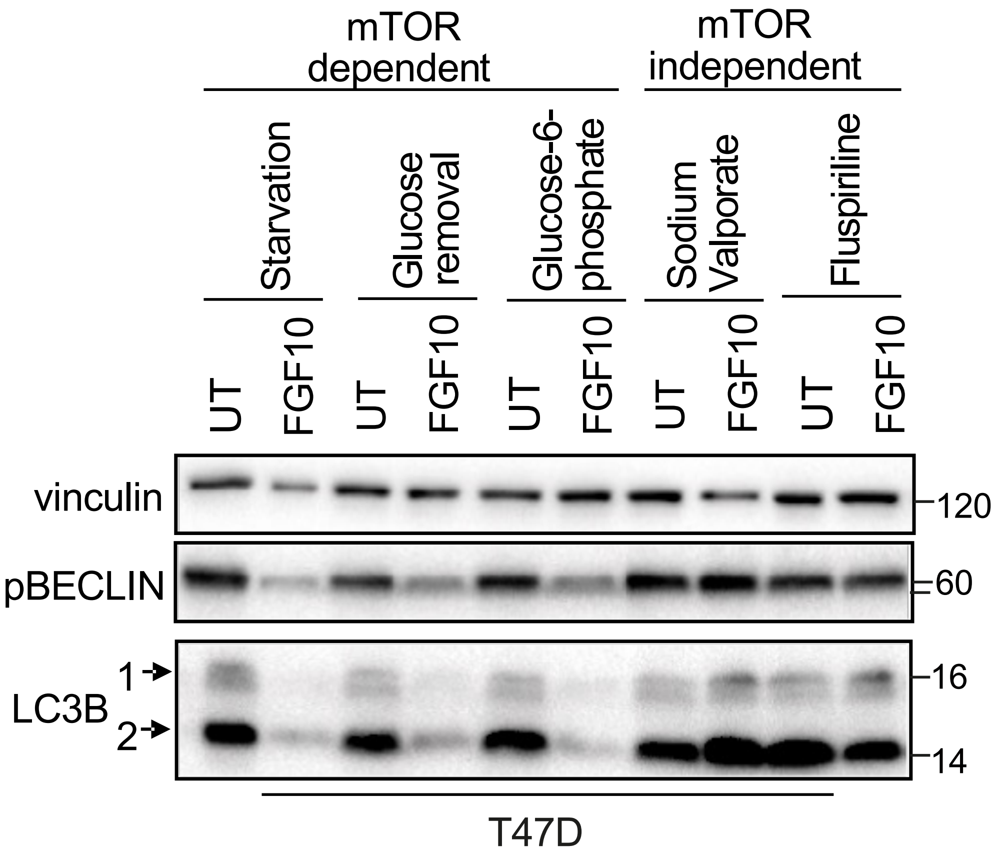

mTOR
dependent
mTOR
independent
Starvation
Glucose
removal
Glucose-6-
phosphate
Sodium
Valporate
Fluspiriline
UT
FGF10
UT
FGF10
UT
FGF10
UT
FGF10
UT
FGF10
vinculin
pBECLIN
LC3B
1
2
120
60
16
14
T47D

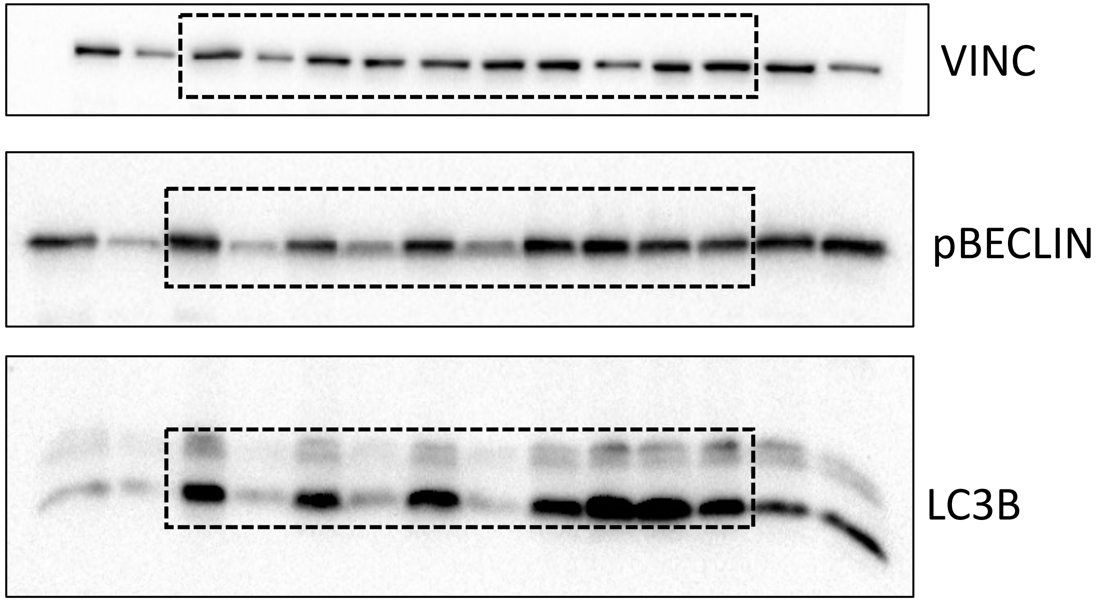

VINC
pBECLIN
LC3B

Figure 6f

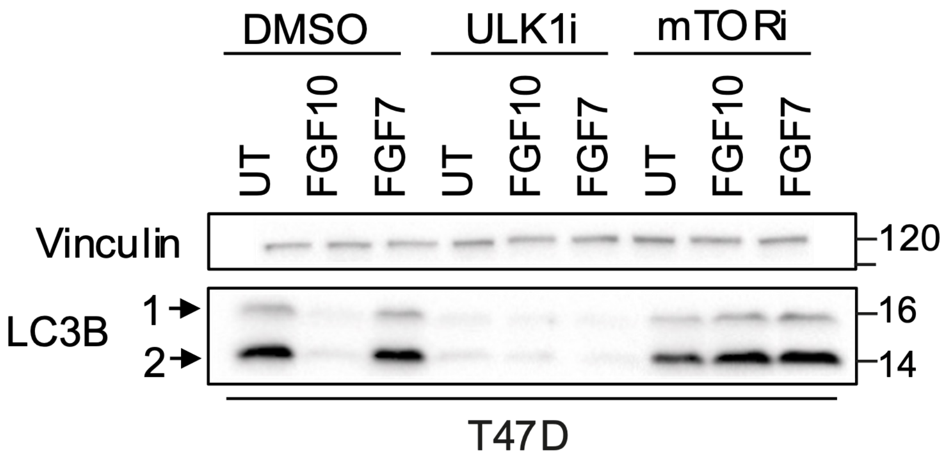
DMSO
ULK1i
mTORi
UT
FGF10
FGF7
UT
FGF10
FGF7
UT
FGF10
FGF7
Vinculin
120
LC3B
1
2
16
14
T47D

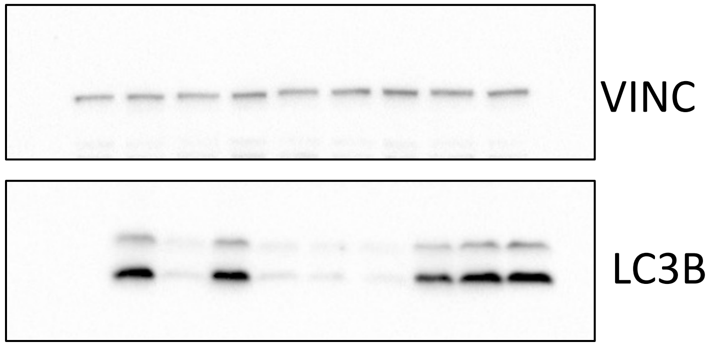
VINC
LC3B

Figure 7a

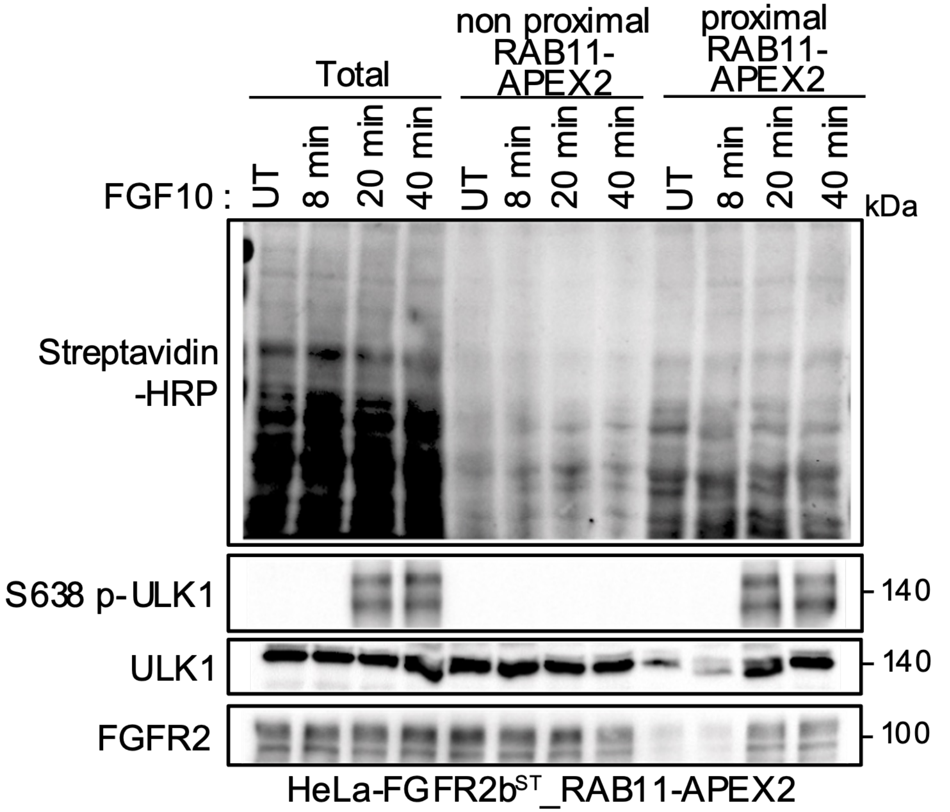
Total
non proximal RAB11-APEX2
proximal RAB11-APEX2
FGF10 :
UT
8 min
20 min
40 min
UT
8 min
20 min
40 min
UT
8 min
20 min
40 min
kDa
Streptavidin -HRP
S638 p-ULK1
140
ULK1
140
FGFR2
100
HeLa-FGFR2bST_RAB11-APEX2

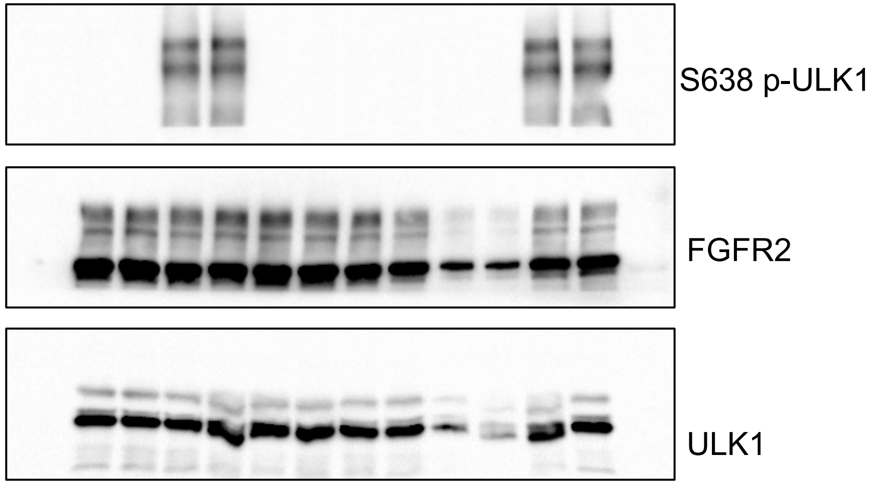
S638 p-ULK1
FGFR2
ULK1

Figure 7b

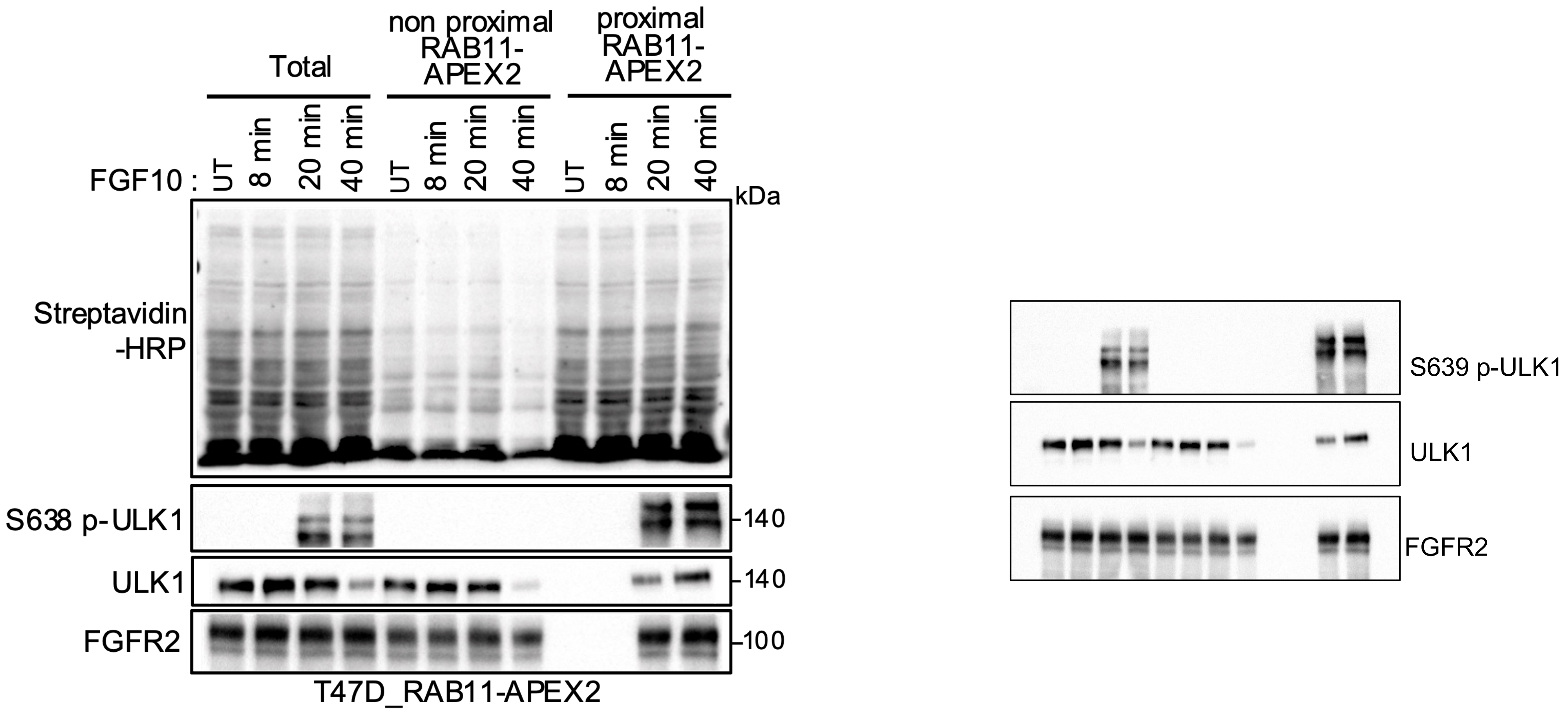
Total
non proximal RAB11-APEX2
proximal RAB11-APEX2
FGF10 :
UT
8 min
20 min
40 min
UT
8 min
20 min
40 min
UT
8 min
20 min
40 min
kDa
Streptavidin -HRP
S638 p-ULK1
ULK1
FGFR2
140
140
100
T47D_RAB11-APEX2
S639 p-ULK1
ULK1
FGFR2

Figure 7f

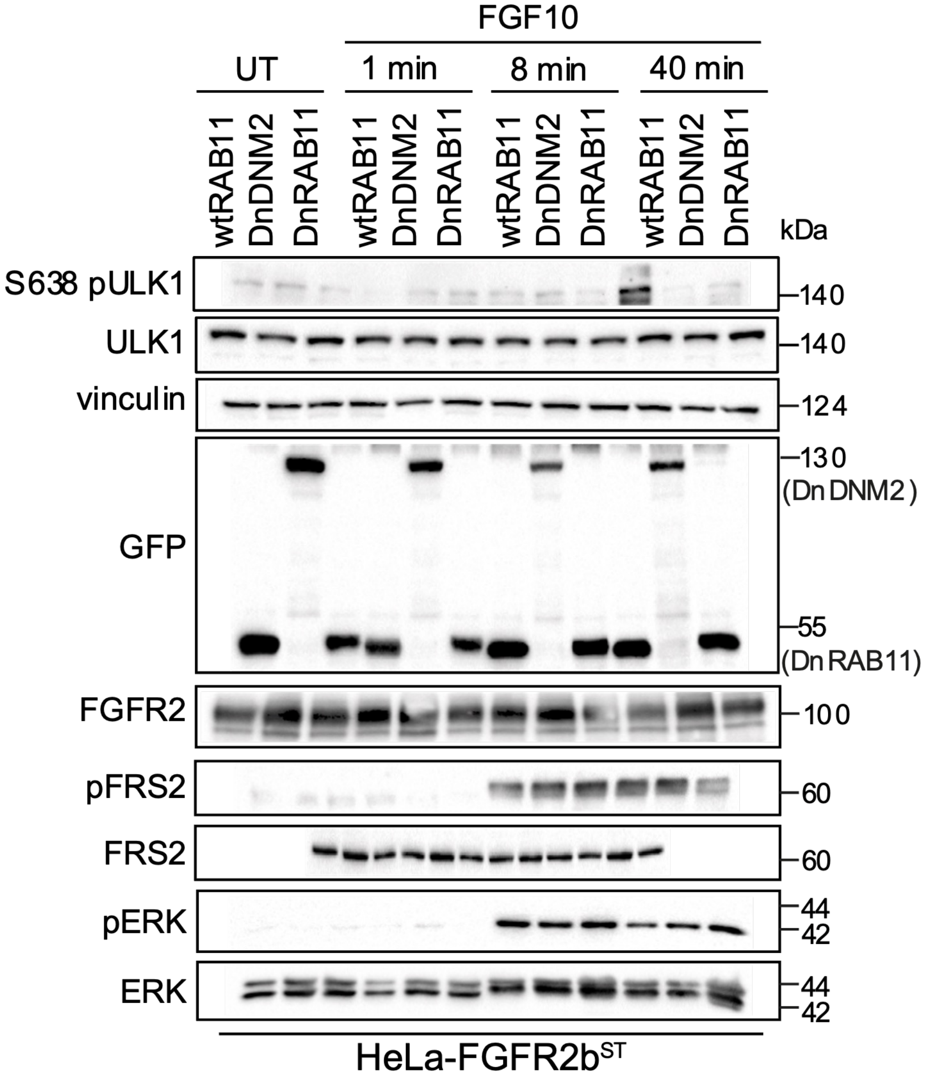
FGF10
UT
1 min
8 min
40 min
wtRAB11
DnDNM2
DnRAB11
wtRAB11
DnDNM2
DnRAB11
wtRAB11
DnDNM2
DnRAB11
wtRAB11
DnDNM2
DnRAB11
kDa
S638 pULK1
140
ULK1
140
vinculin
124
GFP
130
(DnDNM2)
55
(DnRAB11)
FGFR2
100
pFRS2
60
FRS2
60
pERK
44
42
ERK
44
42
HeLa-FGFR2b^ST

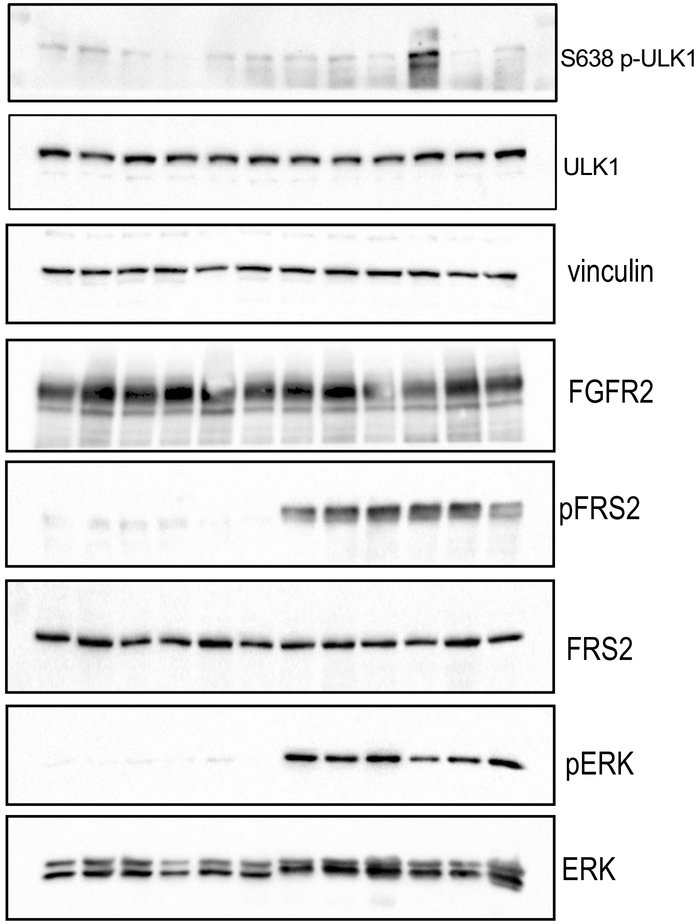
S638 p-ULK1
ULK1
vinculin
FGFR2
pFRS2
FRS2
pERK
ERK

Figure 7g

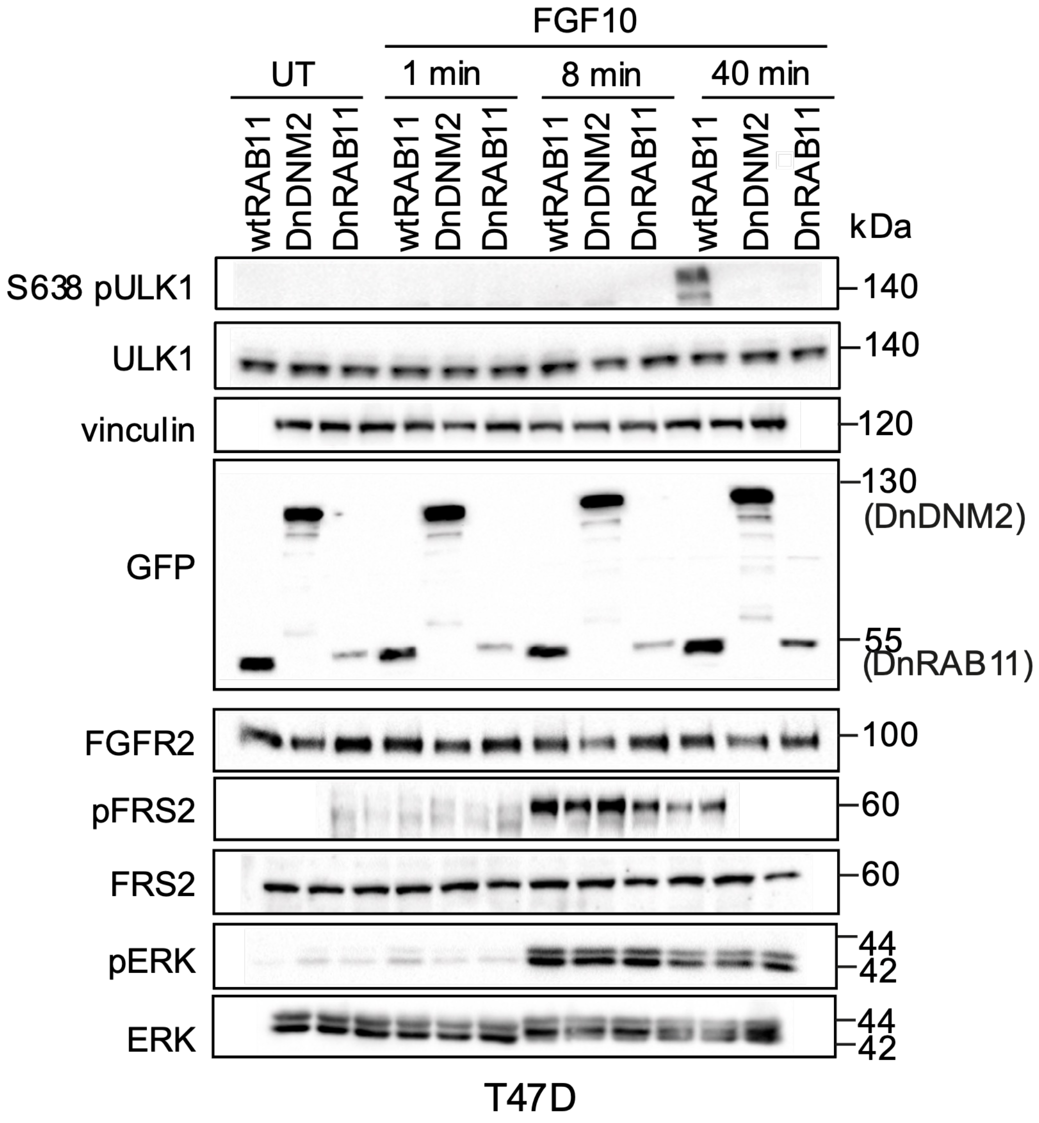
FGF10
UT
1 min
8 min
40 min
wtRAB11
DnDNM2
DnRAB11
kDa
S638 pULK1
ULK1
vinculin
GFP
FGFR2
pFRS2
FRS2
pERK
ERK
140
140
120
130
(DnDNM2)
55
(DnRAB11)
100
60
60
44
42
44
42
T47D

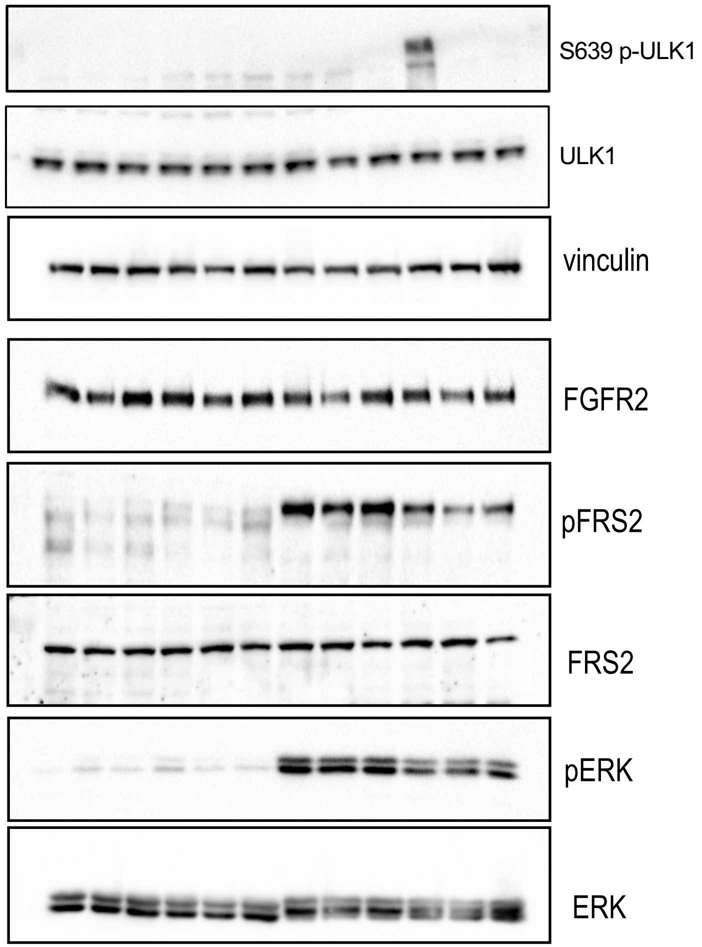
S639 p-ULK1
ULK1
vinculin
FGFR2
pFRS2
FRS2
pERK
ERK

Figure 7k

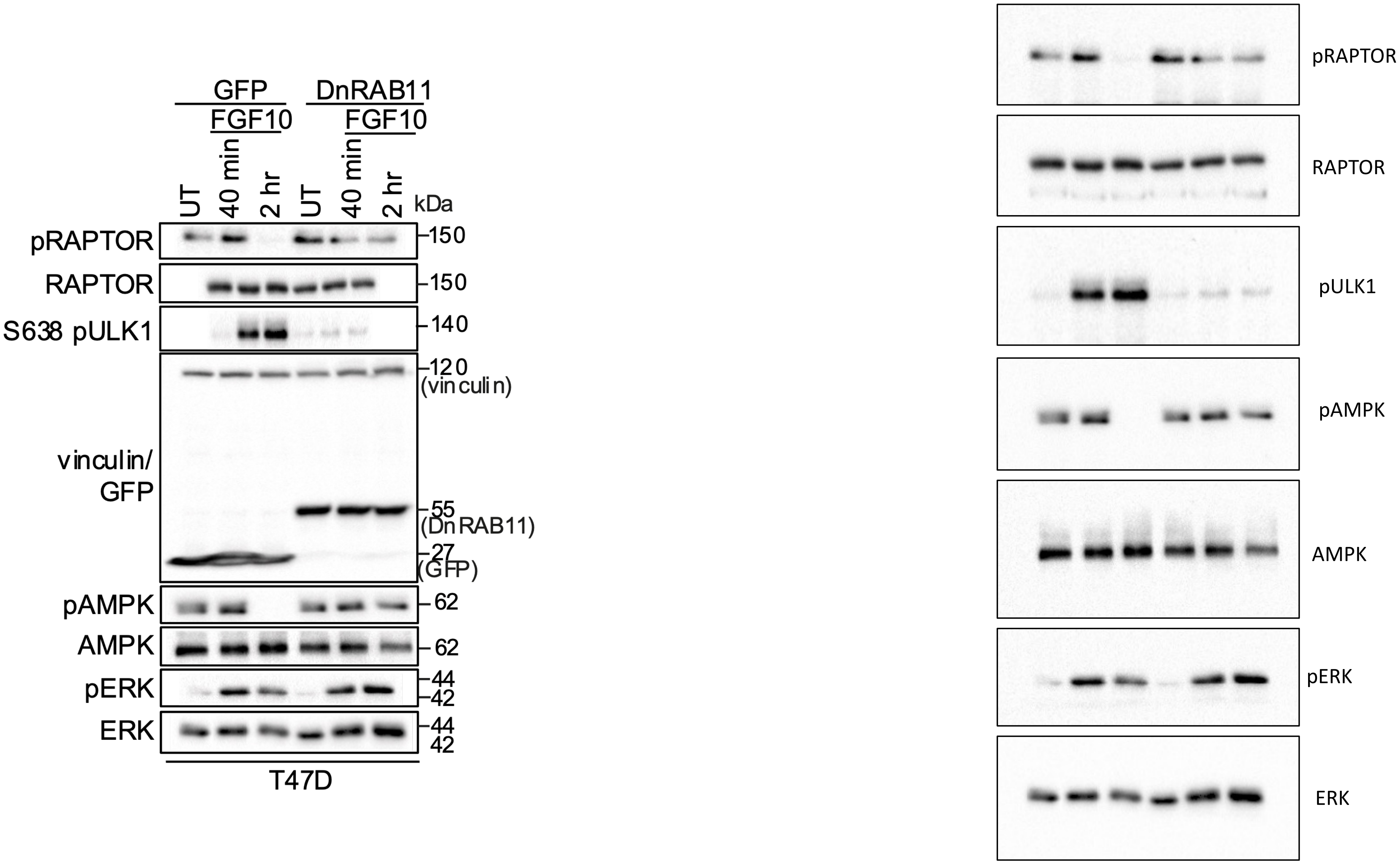
GFP
DnRAB11
FGF10
FGF10
UT
40 min
2 hr
UT
40 min
2 hr
kDa
pRAPTOR
RAPTOR
S638 pULK1
vinculin/
GFP
pAMPK
AMPK
pERK
ERK
150
150
140
120
(vinculin)
55
(DnRAB11)
27
(GFP)
62
62
44
42
44
42
T47D
pRAPTOR
RAPTOR
pULK1
pAMPK
AMPK
pERK
ERK

Figure 8b

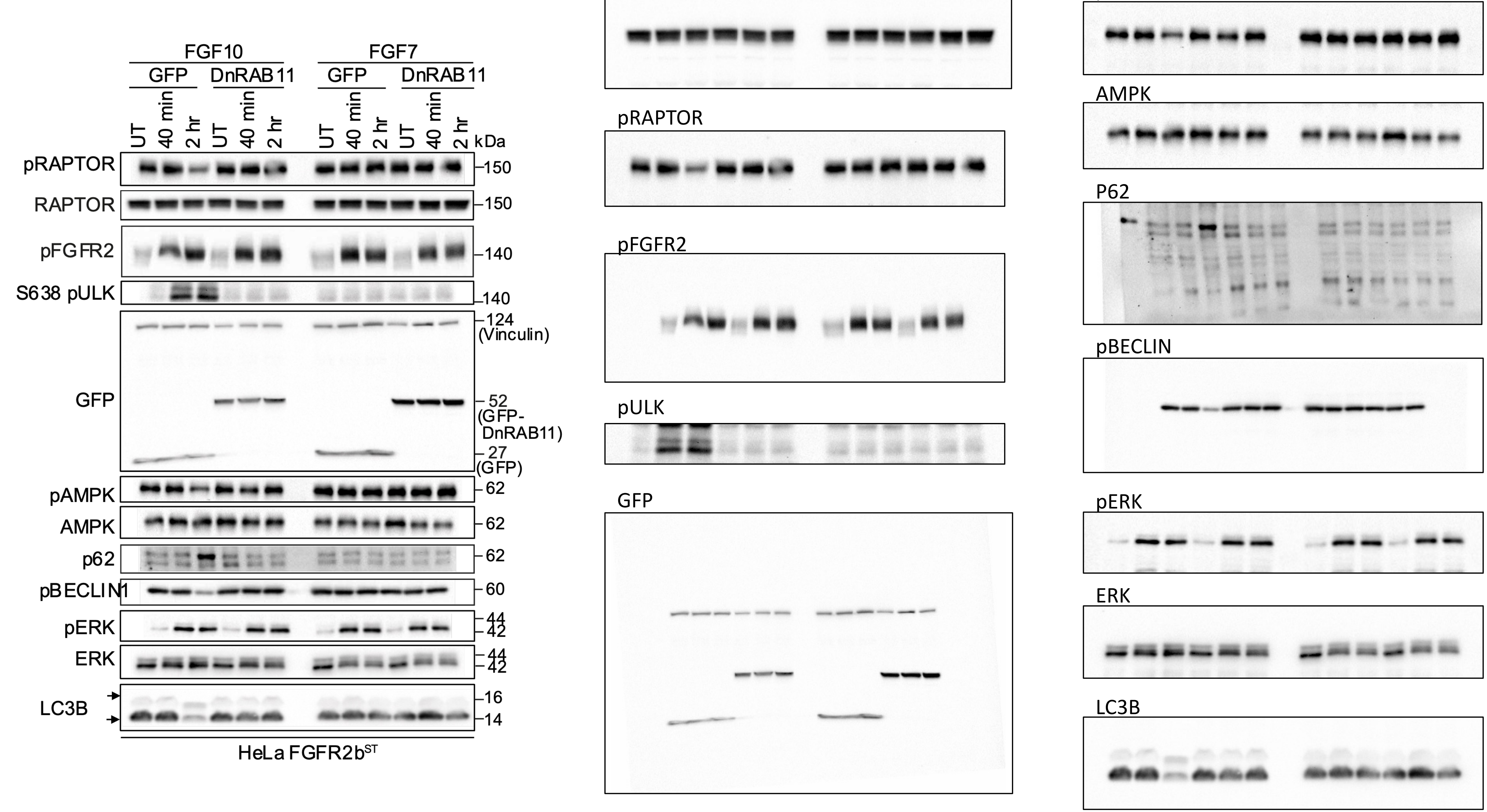
FGF10
FGF7
GFP
DnRAB11
UT
40 min
2 hr
kDa
pRAPTOR
RAPTOR
pFGFR2
S638 pULK
GFP
pAMPK
AMPK
p62
pBECLIN1
pERK
ERK
LC3B
150
140
124 (Vinculin)
52 (GFP-DnRAB11)
27 (GFP)
62
60
44
42
16
14
HeLa FGFR2b^ST
RPTOR
pRAPTOR
pFGFR2
pULK
GFP
pAMPK
AMPK
P62
pBECLIN
pERK
ERK
LC3B

Supplementary Figure 1a

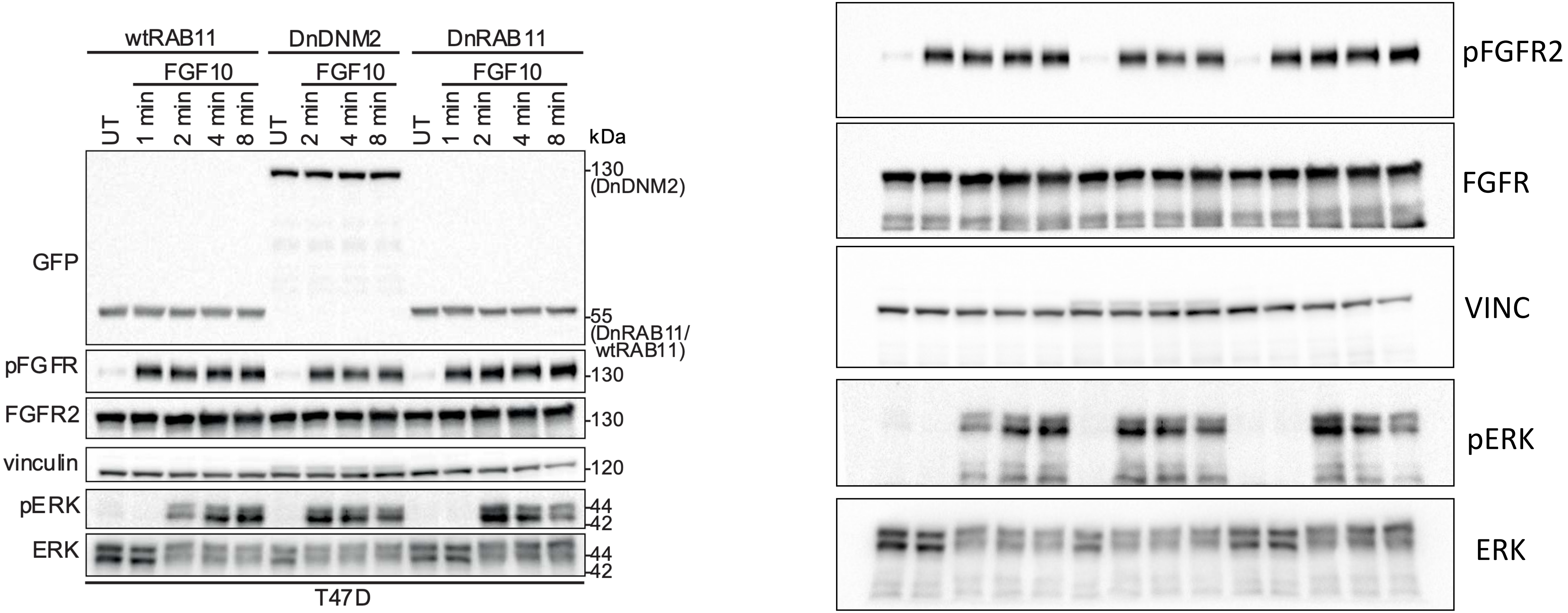

Supplementary Figure 1b

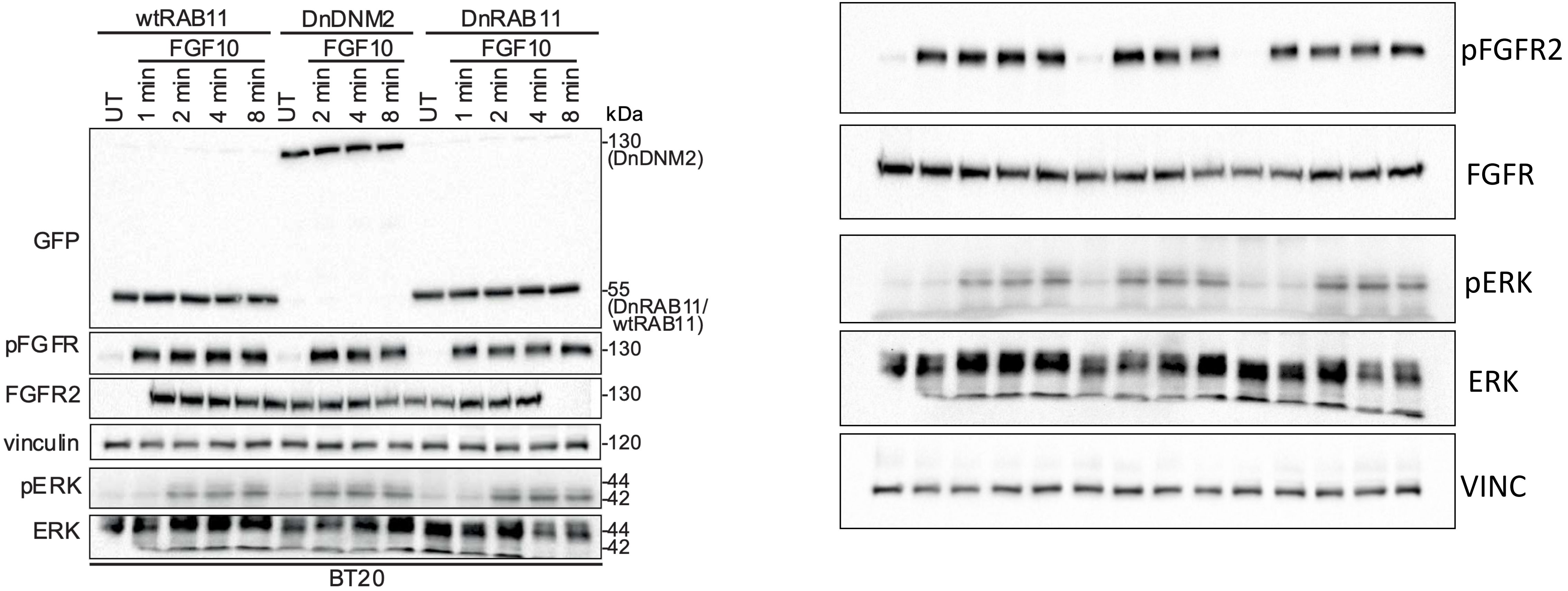

Supplementary Figure 2i

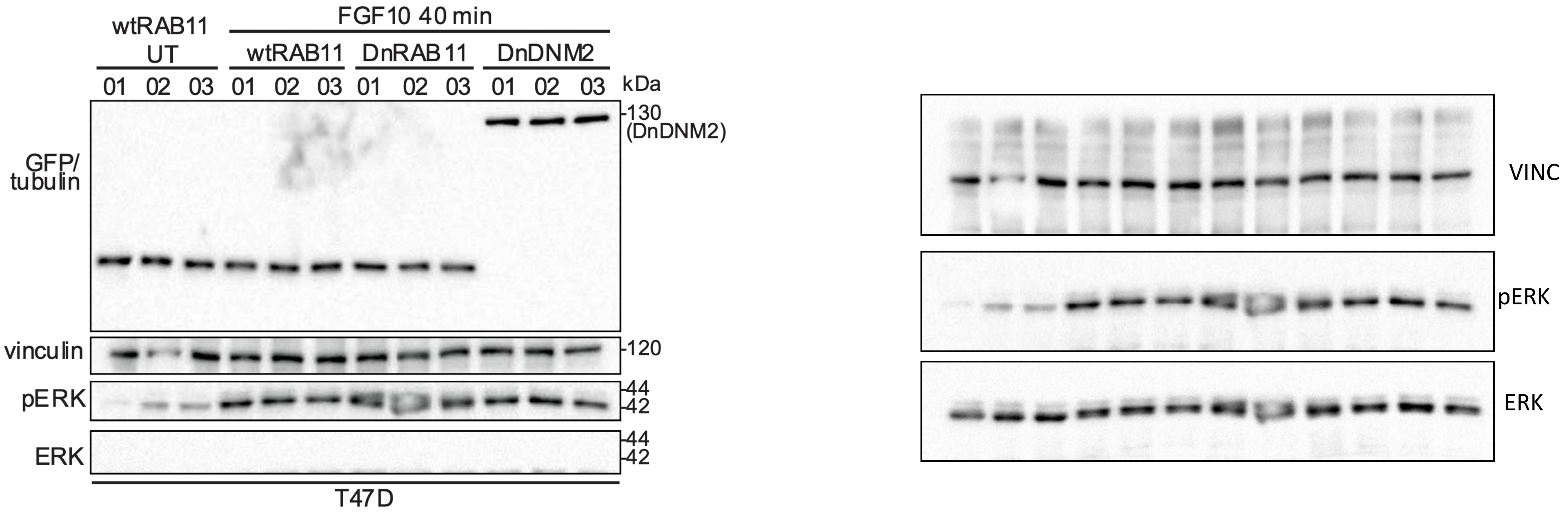

Supplementary Figure 3a

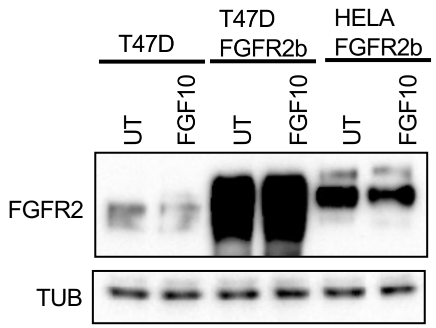

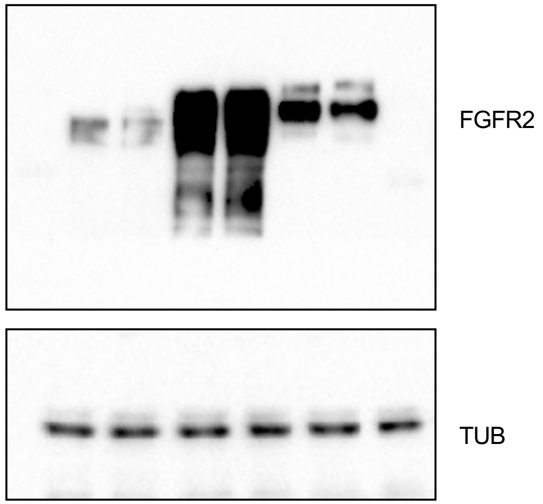

Supplementary Figure 4a

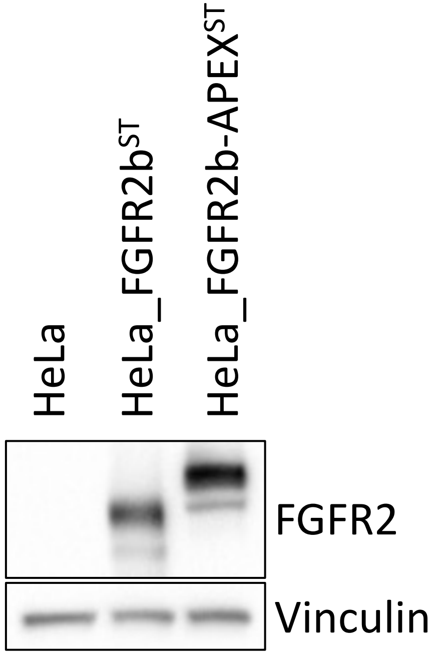

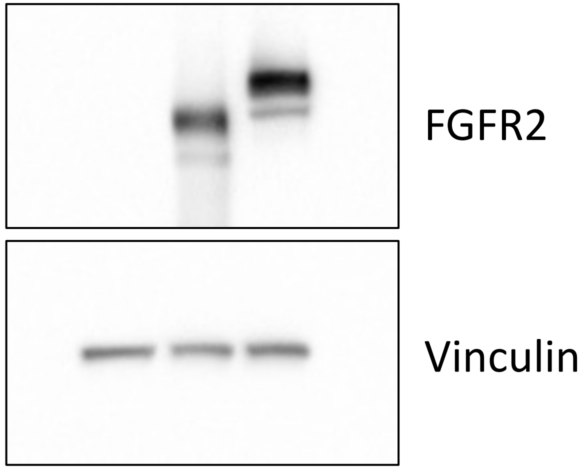

Supplementary Figure 4d

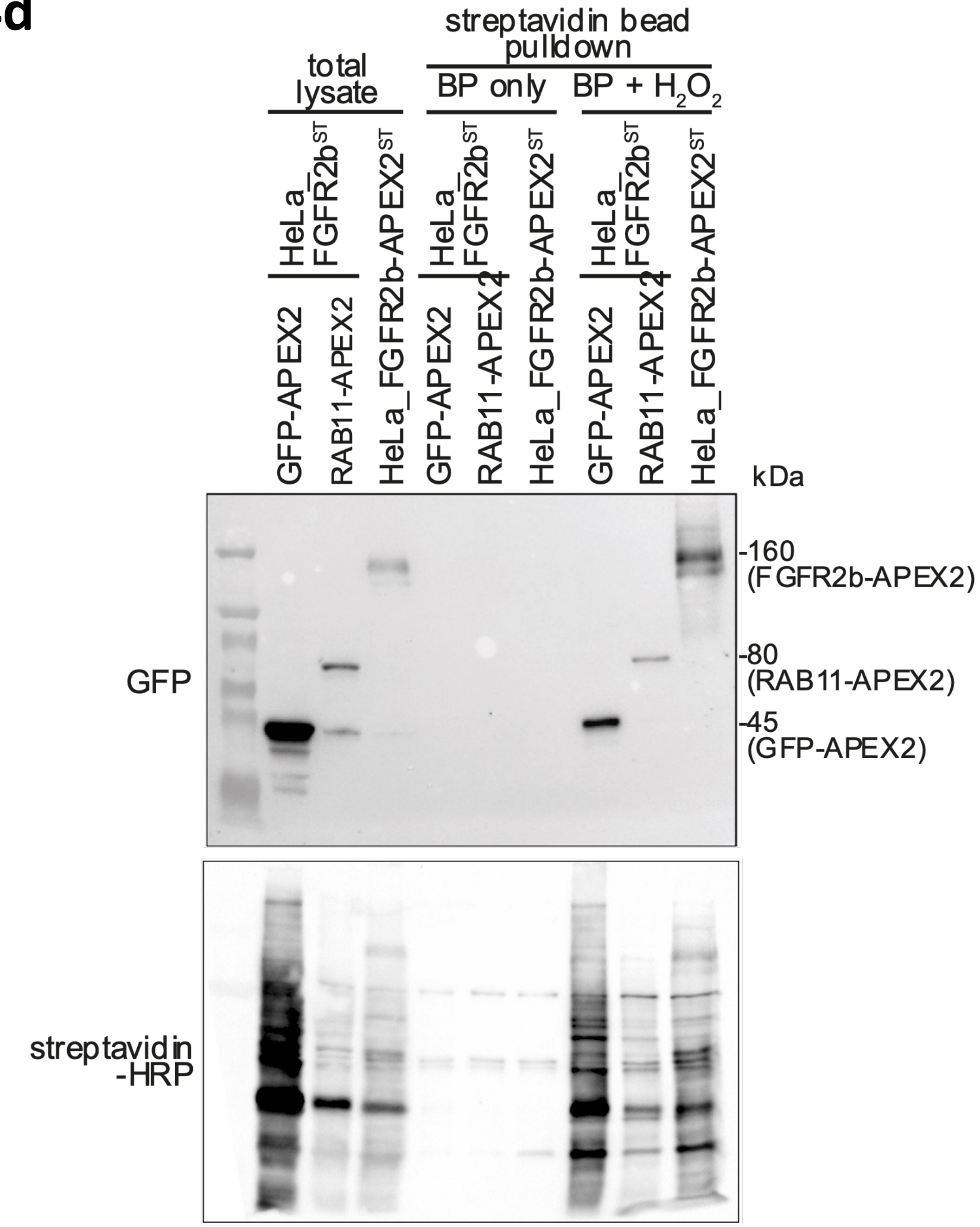

Supplementary Figure 4e

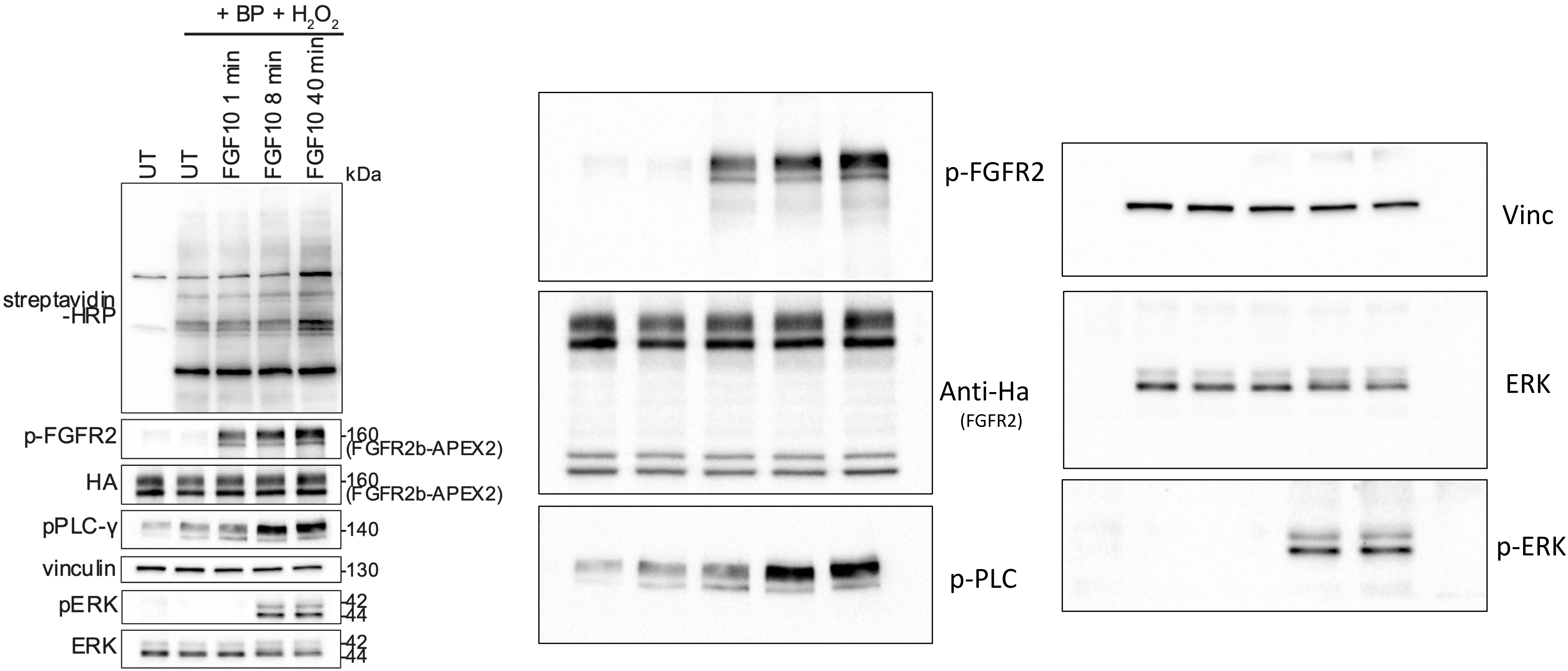

Supplementary Figure 4f

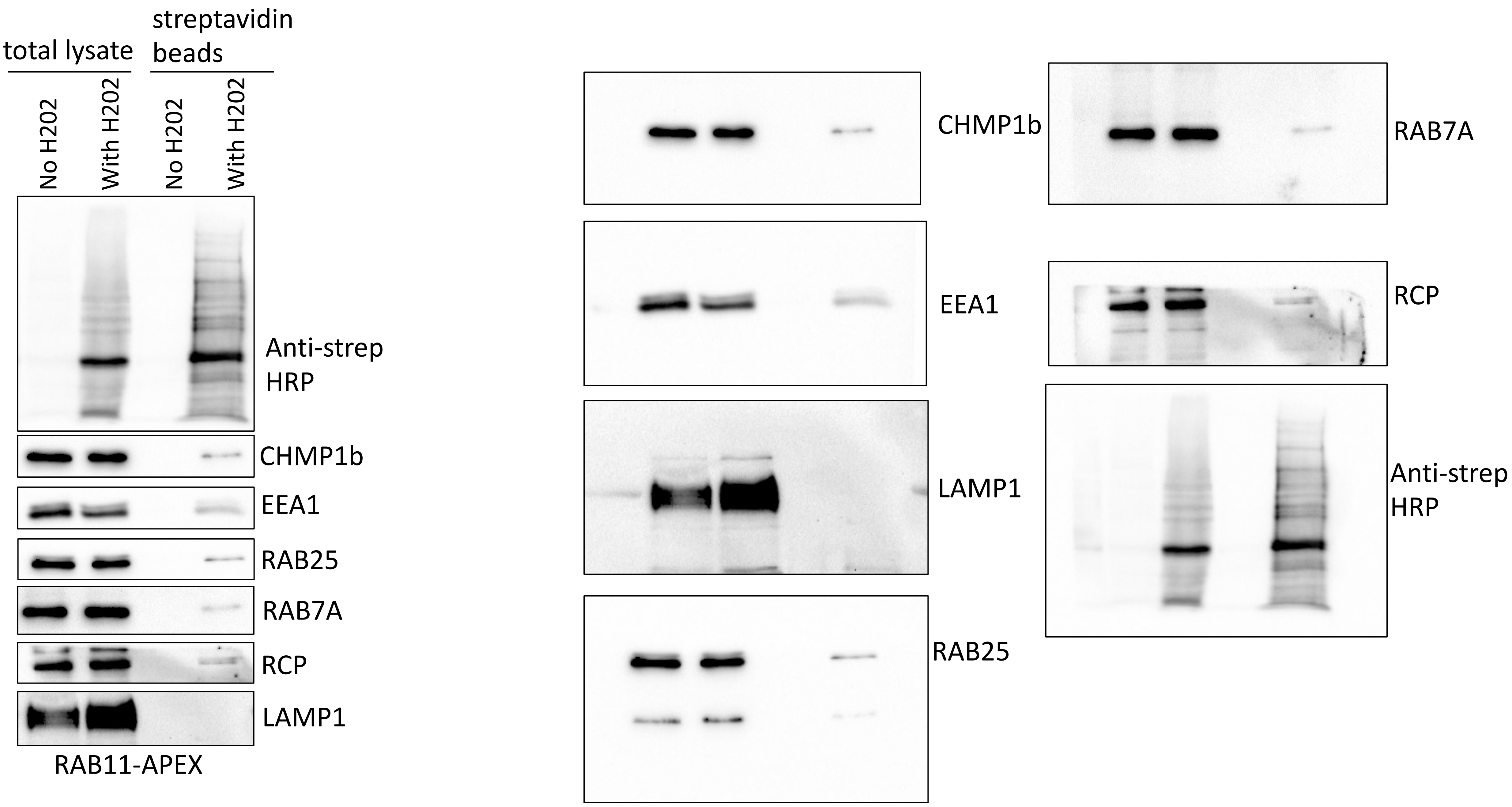

Supplementary Figure 7a

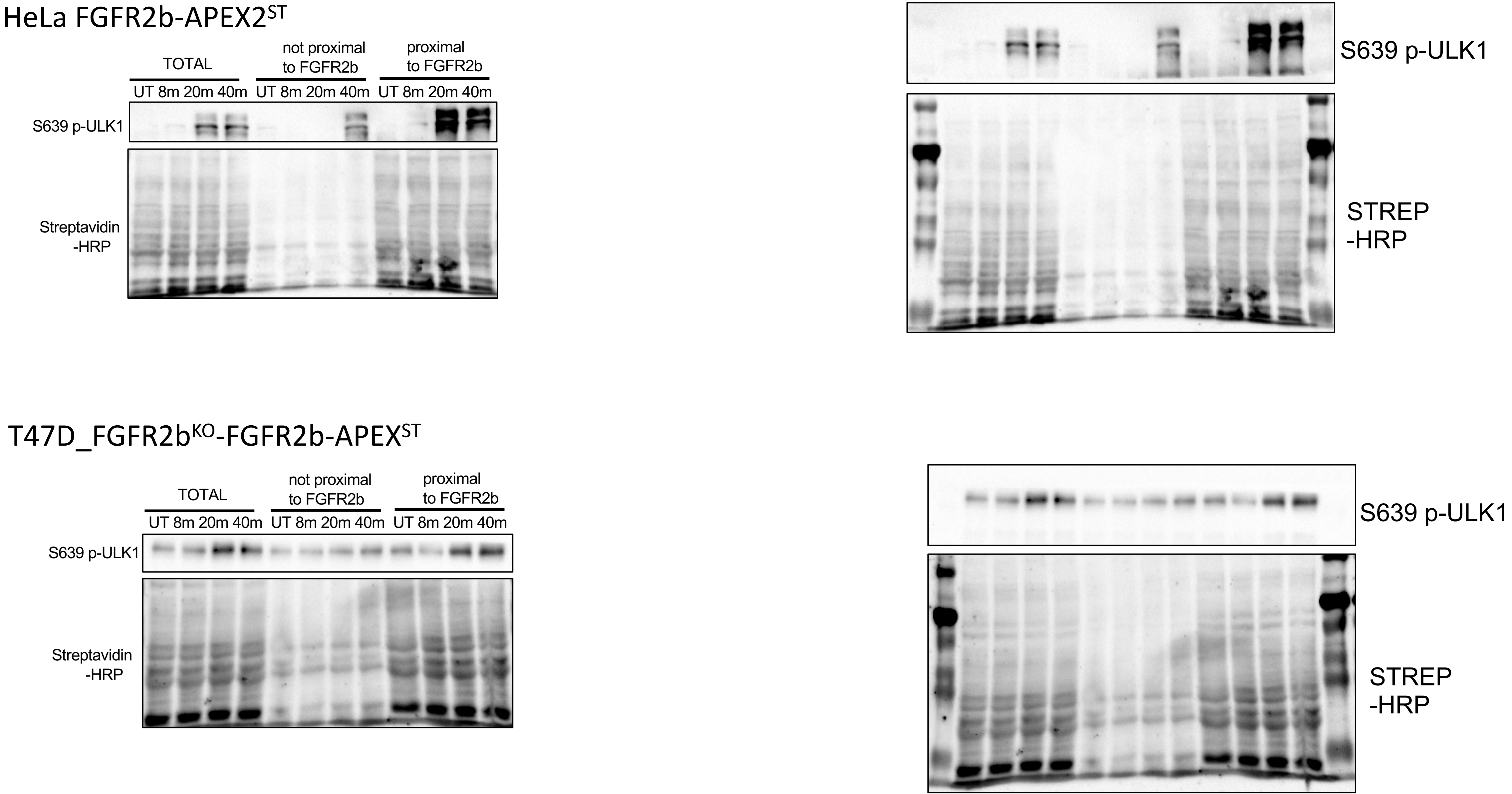

Supplementary Figure 7c

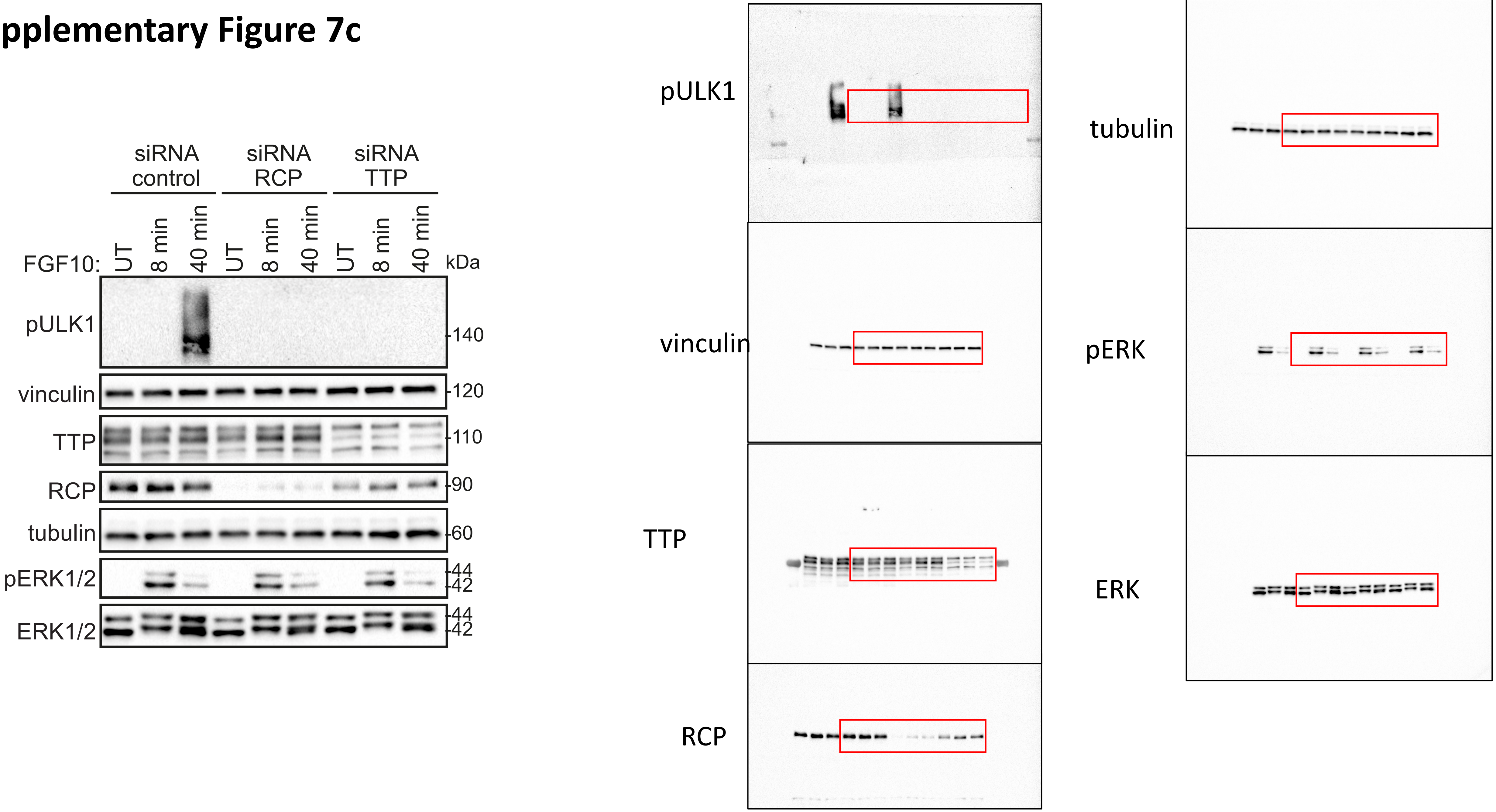

Supplementary Figure 7d

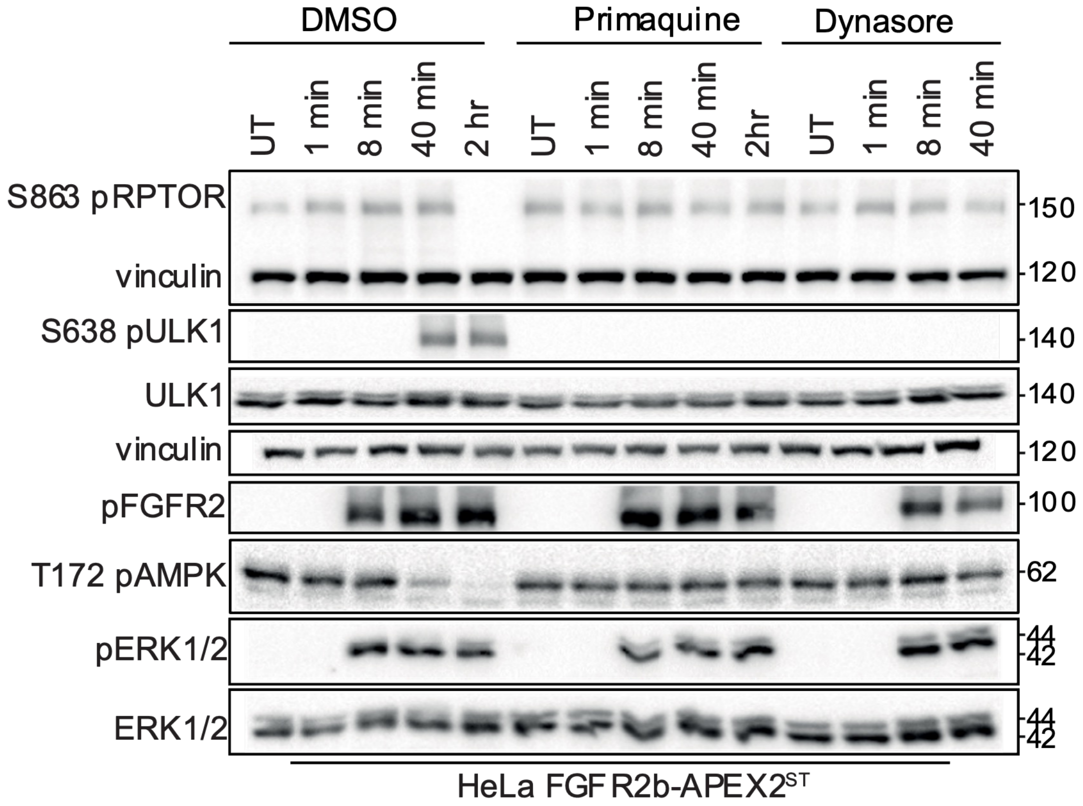

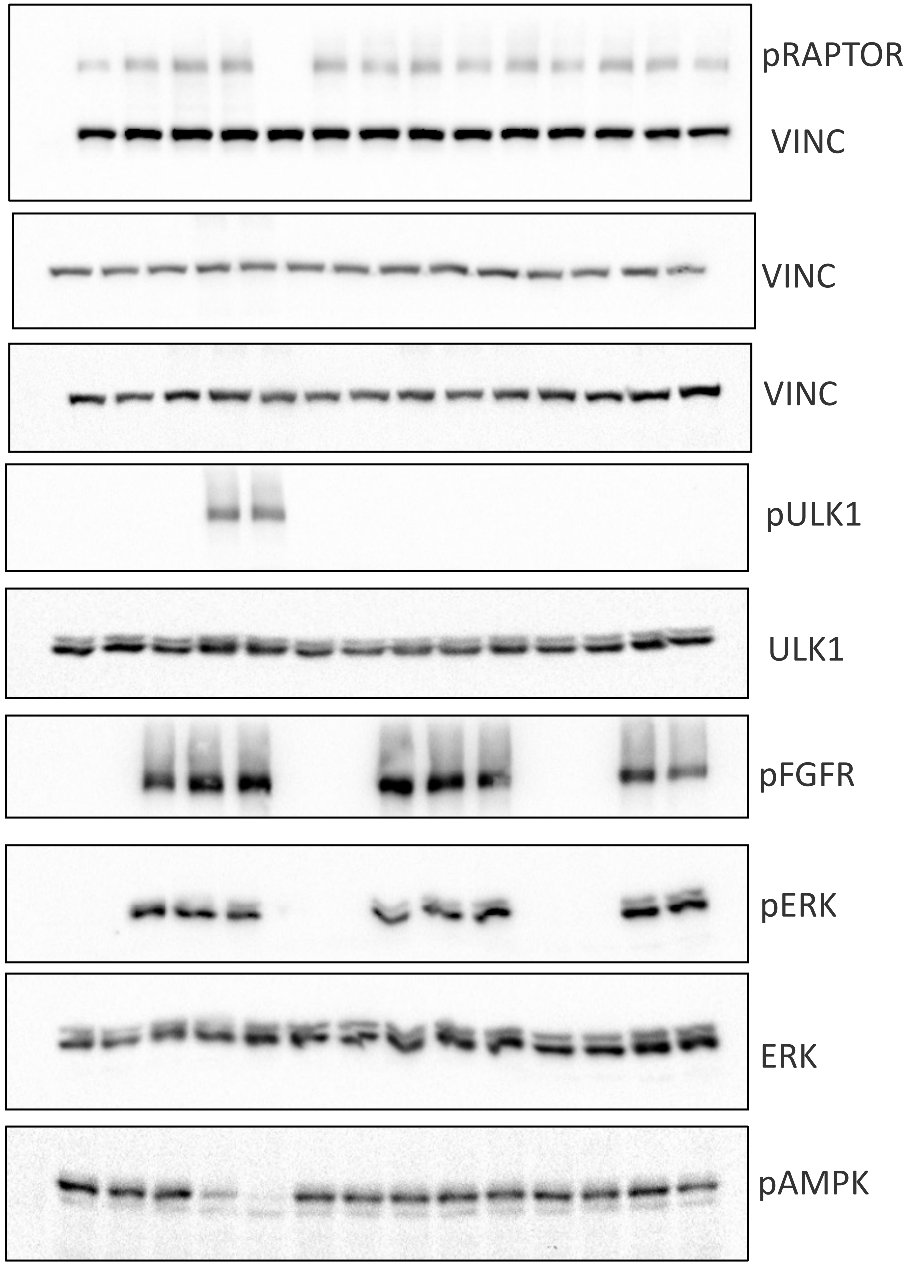

Supplementary Figure 7e

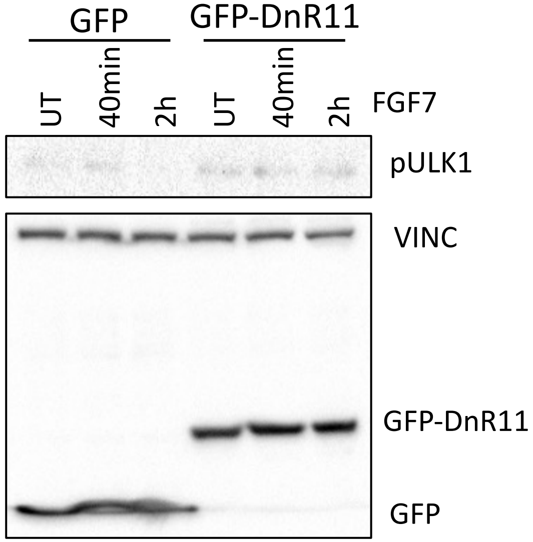

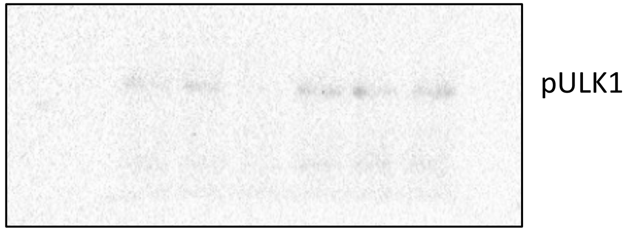

Supplementary Figure 8d

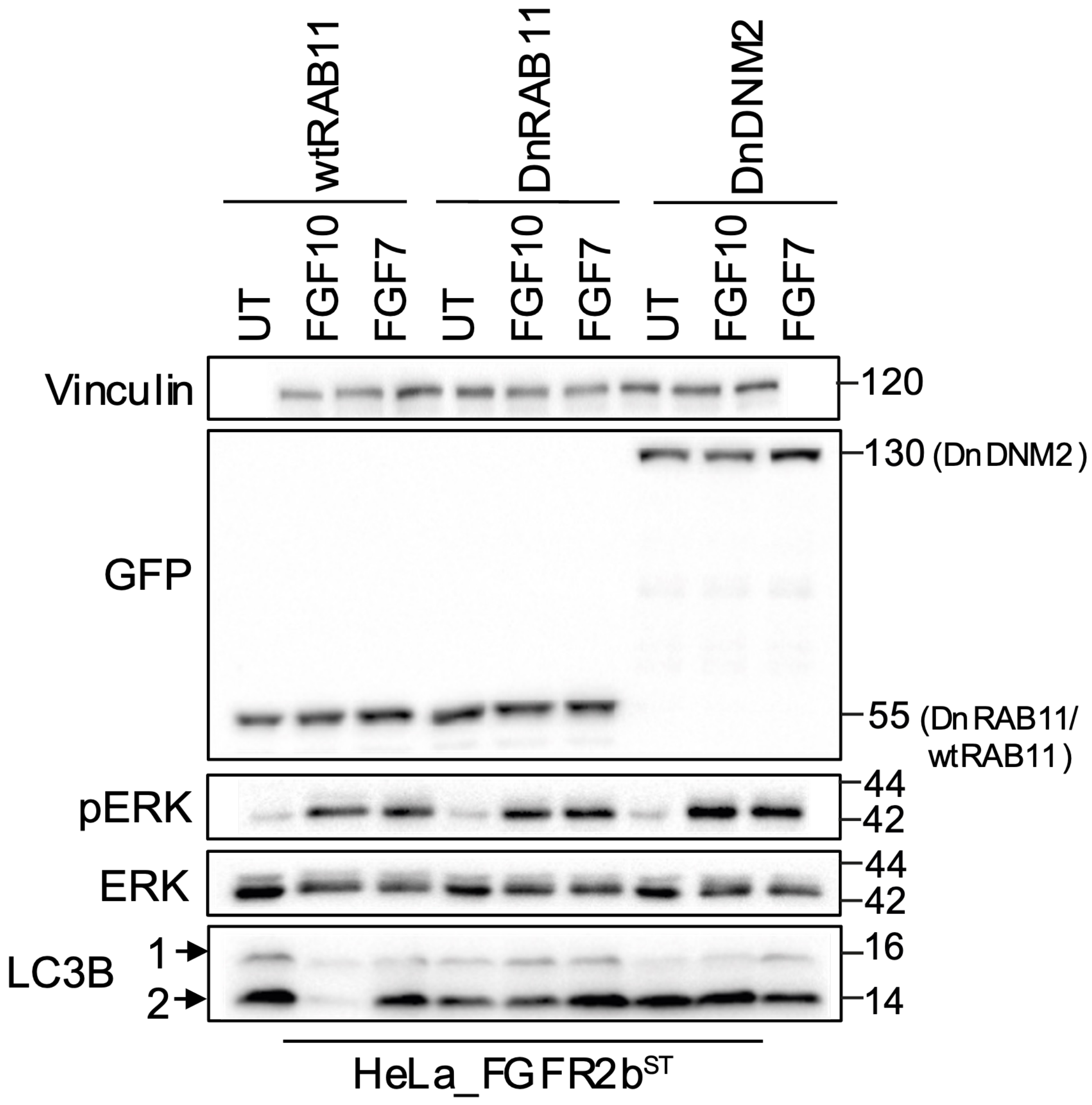

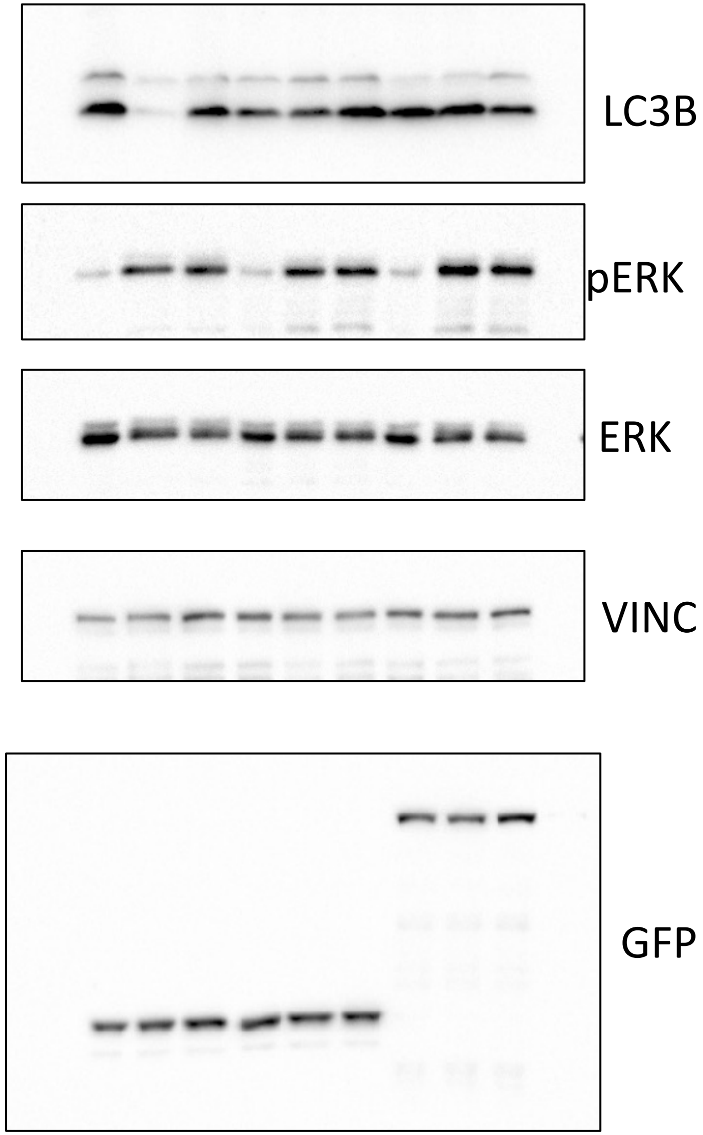

Supplementary Figure 8e

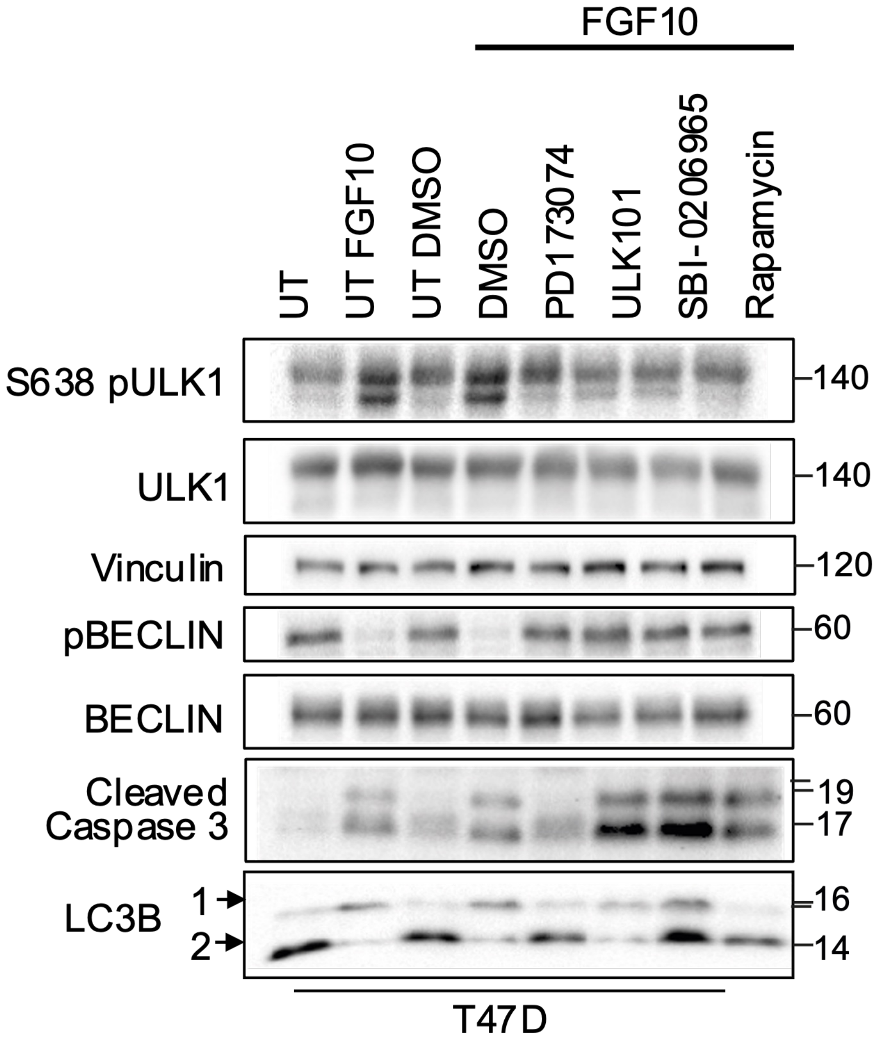

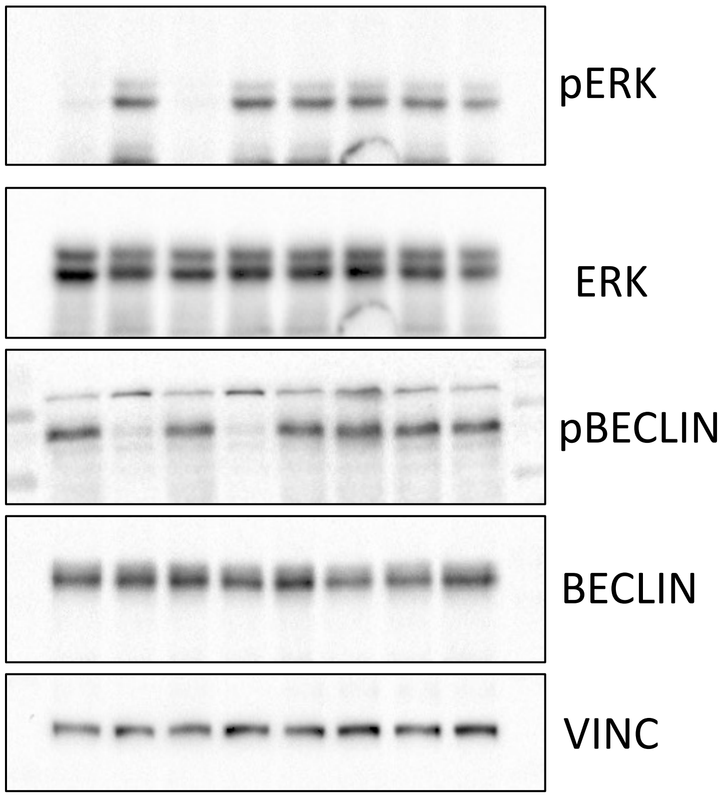

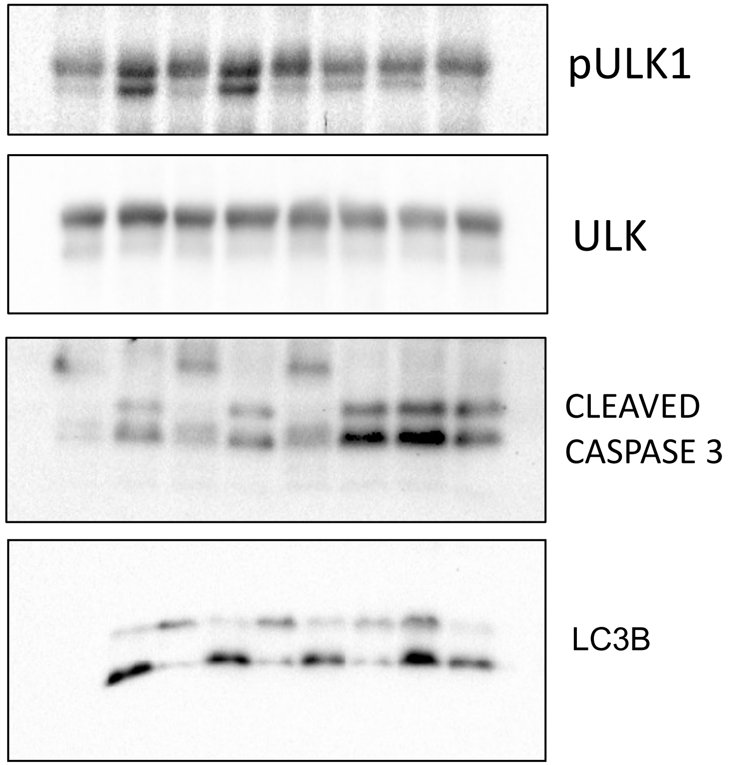

Supplement: Supplementary file 11 — Source Data [file 41467_2022_34298_MOESM11_ESM.zip › WesternBlots_Watson_Ferguson.pdf]
